# Engineering of Asymmetric Dual-Atom Sites for Effective Oxygen Electrocatalysis

**DOI:** 10.1007/s40820-026-02155-2

**Published:** 2026-04-07

**Authors:** Dongping Xue, Yu Zhao, Jianliang Cao, Yan Wang

**Affiliations:** 1 College of Chemistry and Chemical Engineering, Henan Polytechnic University, Jiaozuo, 454003 People’s Republic of China; 2 College of Safety Science and Engineering, Henan Polytechnic University, Jiaozuo, 454003 People’s Republic of China

**Keywords:** Asymmetric dual-atom sites, Synergetic effect, Spin configuration, Oxygen electrocatalysis

## Abstract

Asymmetric dual-atom site catalysts (ADASCs) inherit the high atomic utilization of single-atom site catalysts and synergistic effects of symmetric dual-atom site catalysts, while uniquely integrating the asymmetry of heteronuclear metal centers and asymmetric coordination environments. This structural merit endows them with tunable electronic configurations and optimized reaction kinetics, breaking conventional catalysts’ inherent limitations to boost catalytic performance significantly. Despite existing reviews on dual-atom site catalysts for electrocatalysis, a comprehensive asymmetry-focused framework to elucidate ADASCs’ catalytic behaviors remains elusive. This review summarizes the multi-dimensional regulation mechanisms of ADASCs. Structurally, heteronuclear metal synergy and bridging ligand co-anchoring suppress single-atom agglomeration. Electronically, *d*-band center modulation, spin coupling, and orbital hybridization optimize intermediate adsorption energies. Subsequently, the strategies for constructing asymmetric structures are elaborated, including the design of heteronuclear metal centers and the regulation of asymmetric coordination environments via nonmetallic atom engineering. Furthermore, the prominent advantages of ADASCs in oxygen electrocatalysis are highlighted as their asymmetric configurations can break the linear scaling relationship of intermediate adsorption, and optimize the reaction pathway as well as the adsorption behaviors of key intermediates. Finally, the current challenges and opportunities of ADASCs are discussed, providing valuable insights for the development of high-performance energy conversion devices.

## Highlights

- The multi-dimensional performance optimization mechanisms of asymmetric dual-atom site catalysts (ADASCs) are summarized, laying a fundamental framework for understanding their catalytic behaviors.
- Various strategies for constructing ADASCs are elaborated, with a focus on heteronuclear metal centers and heteroatom doping engineering to disrupt the symmetry between electric fields and structures.
- The applications of ADASCs in oxygen electrocatalysis are systematically summarized, and the structure–performance relationship between asymmetric dual-site configurations and catalytic reactions is revealed.

## Graphical abstract

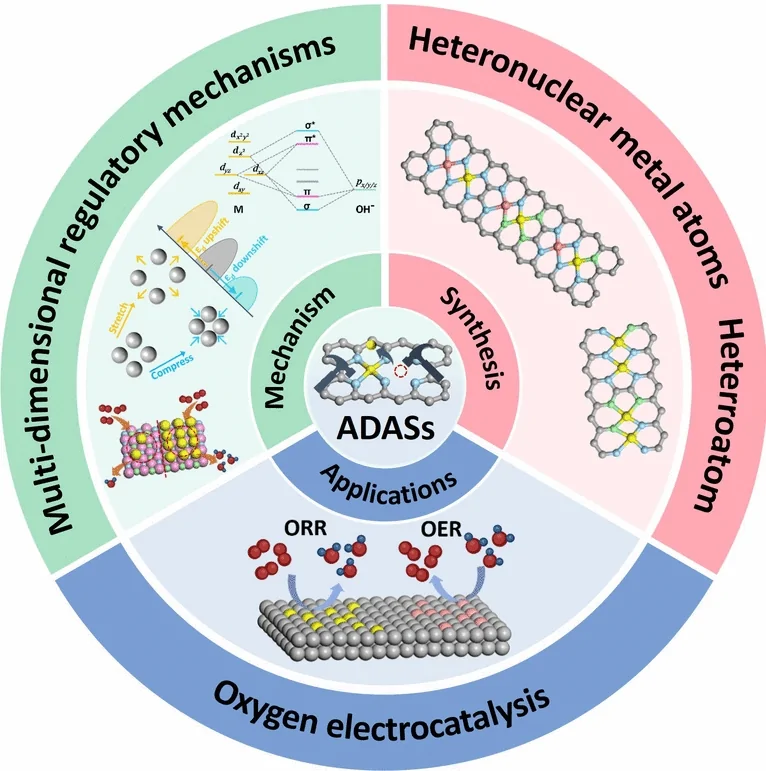

## Introduction

Driven by the global energy crisis and the goal of "carbon neutrality," the development of efficient, clean, and sustainable energy conversion technologies has become a core demand in addressing environmental issues and transforming energy structures [1–5]. Advanced energy conversion devices such as fuel cells, rechargeable metal–air batteries, and proton exchange membrane water electrolyzers are regarded as key components of the next-generation energy systems due to their high energy density and low pollution emissions [6–11]. However, these devices are limited by the sluggish intrinsic kinetics of their core half-reactions, namely the oxygen reduction reaction (ORR) and the oxygen evolution reaction (OER), which lead to reduced energy conversion efficiency and significant overpotential losses [12–14]. Therefore, high-performance electrocatalysts are needed to break through this bottleneck.

**Fig. 1:**
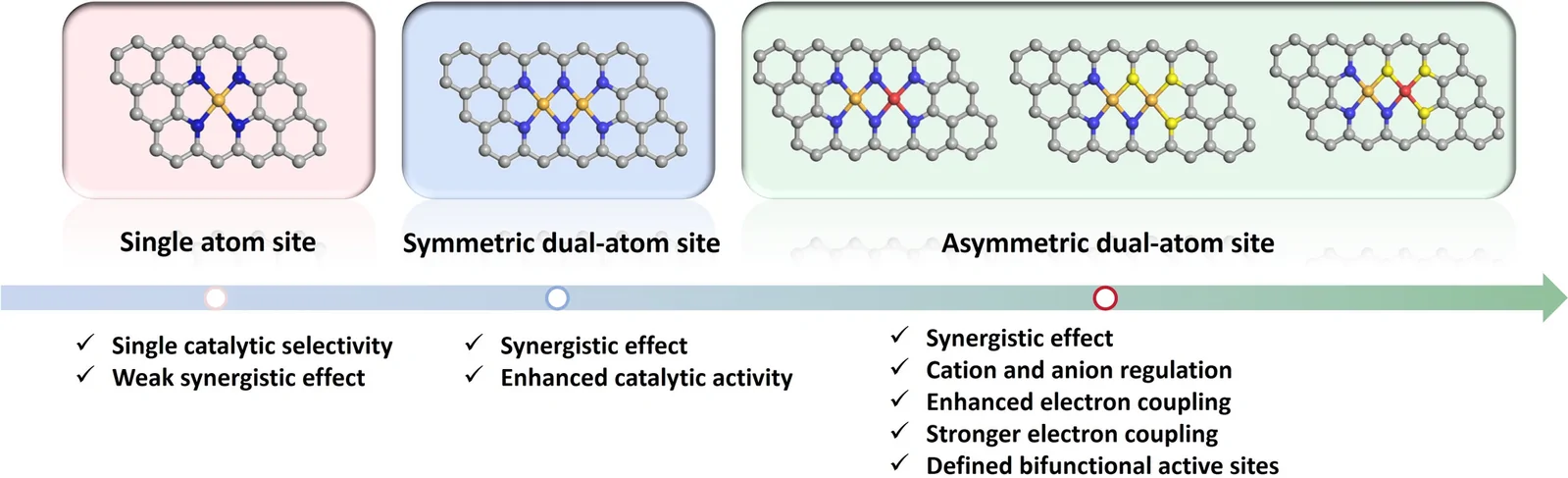
Classification and characteristics of SACs, SDASCs, and ADASCs

As shown in Fig. 1, single-atom site catalysts (SASCs) have been widely applied in cutting-edge electrochemical energy conversion owing to their nearly 100% atomic utilization efficiency, unique electronic structure, and well-defined active sites [15–17]. Among them, the M–N_4_ symmetric structure, with local planar D_4_h symmetry, endows SASCs with unique physicochemical properties and significant potential for enhancing the kinetics of electrocatalytic reactions [17, 18]. Although SASCs have achieved remarkable progress in boosting the efficiency of electrocatalytic reactions, several challenges still hinder their further development to meet practical application demands, including low metal loading, weak interactions between isolated metal atoms, insufficient adsorption sites, and sluggish electron transfer associated with the symmetric M–N_4_ coordination structure [19]. As an extension of SASCs, dual-atom site catalysts (DASCs) not only inherit the advantages of SASCs but also possess potential merits of geometric ensemble effect and electronic ligand effect in catalysts [20–23]. Nevertheless, DASCs with homonuclear metal centers and symmetric coordination environments still cannot break through the constraints of the linear scaling relationship (LSR) of multi-step reaction intermediates. This limitation leads to sub-optimal adsorption of reaction intermediates, primarily because the uniform electronic environment around the active sites fails to interact sufficiently with diverse intermediate species [24]. To fully unlock the potential of DASs in multi-step catalytic reactions, asymmetric dual-atom site catalysts (ADASCs) have emerged prominently, as they can induce inhomogeneous electric or stress fields to maximize functionality via multiple synergistic effects [25–28]. Currently, the supports employed for constructing ADASCs are primarily categorized into carbon materials [16, 29, 30] (e.g., carbon nanotubes, graphene, MOF-derived porous carbon), metal oxides [31], sulfides/phosphides [32, 33], and layered two-dimensional (2D) materials (e.g., graphitic carbon nitride (g-C_3_N_4_), MXenes, layered double hydroxides) [34, 35], among which carbon materials dominate the fabrication of ADASCs due to their unique advantages in structure, electronics, synthesis, and cost-effectiveness: (1) Superior electronic conductivity and tunable electronic structure [36]. Carbon supports exhibit high electrical conductivity, which facilitates interfacial electron transfer and reduces the charge transfer resistance of catalytic reactions. Moreover, heteroatom doping endows the carbon support surface with abundant coordination sites, enabling precise modulation of the electronic configuration of bimetallic centers and optimization of the adsorption–desorption kinetics of reaction intermediates. (2) Porous architecture and high specific surface area, favoring active site dispersion and mass transport [37]. The porous structure (micropores and mesopores) of carbon supports provides a large specific surface area, which not only enhances the dispersion of bimetallic sites and suppresses atomic agglomeration but also promotes the mass transport of electrolytes and gaseous reactants, thereby boosting the overall catalytic efficiency. (3) Facile and precisely controllable synthesis protocols [38]. The preparation and modification of carbon supports rely on well-established techniques (e.g., pyrolysis, template-assisted synthesis, impregnation). By regulating parameters such as precursor type and pyrolysis temperature, the interatomic spacing, coordination environment, and loading capacity of bimetallic sites can be precisely manipulated. Meanwhile, the interactions between carbon supports and bimetallic centers are readily characterized, facilitating in-depth mechanistic investigations. (4) Excellent compatibility and cost-effectiveness [39]. Carbon supports possess high chemical stability, effectively resisting phase transformation and corrosion even under acidic, alkaline, or high-potential conditions. Furthermore, they feature broad feedstock sources (e.g., biomass, polymers) and significantly lower production costs compared to emerging supports like MXenes, rendering them more suitable for large-scale manufacturing. (5) Strong compatibility with practical devices [40]. In practical devices such as fuel cells and electrolyzers, carbon supports can be directly integrated with electrode slurries to ensure robust interfacial contact without additional modification. In contrast, oxide and sulfide supports typically require conductivity-enhancing modification to meet the performance requirements of device applications. Accordingly, this review presents a systematic overview focusing on the modulation, construction, catalytic mechanisms, and performance optimization of carbon-supported ADASCs.

**Fig. 2:**
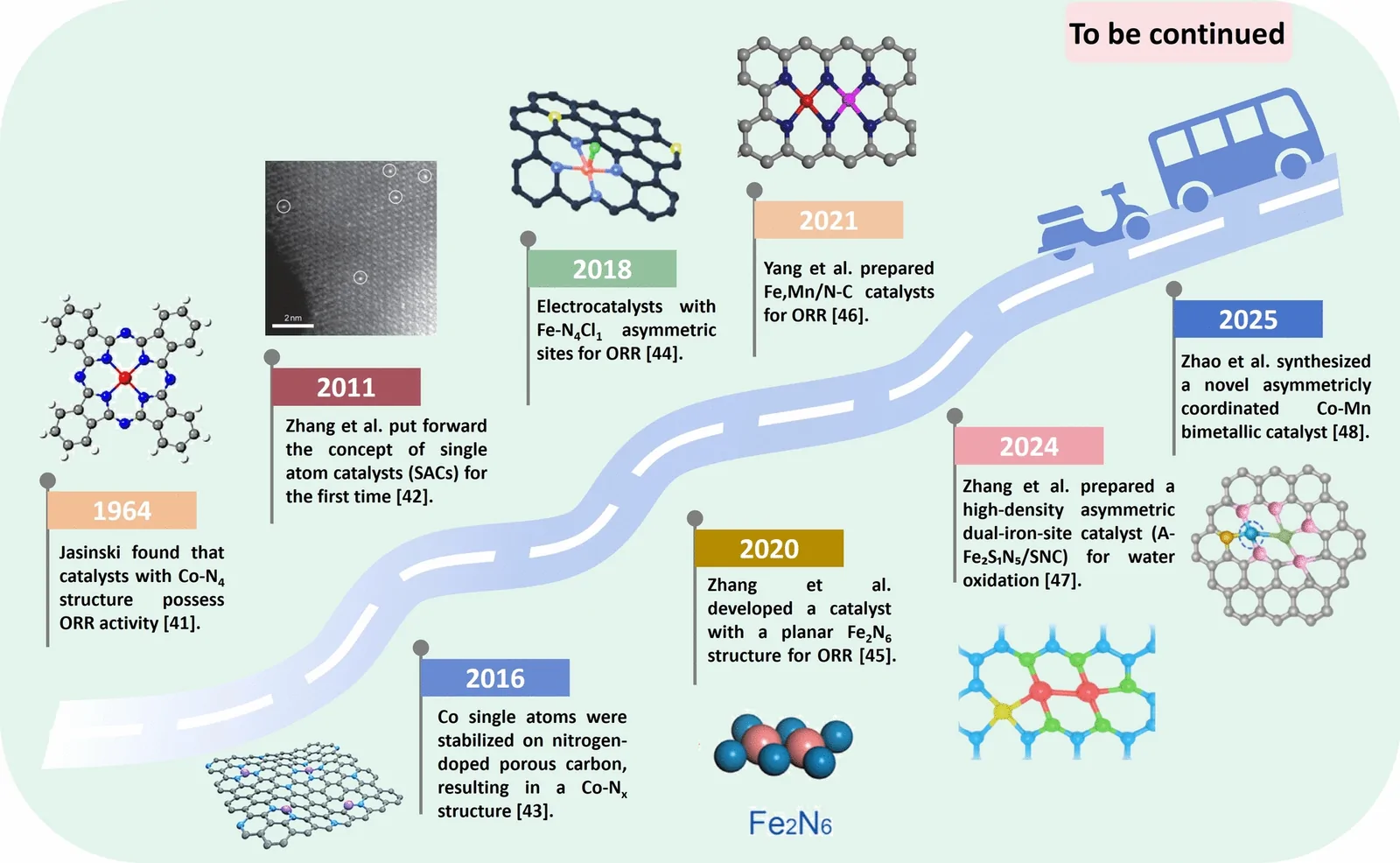
A brief history of the development and the latest progress of ADASCs

In recent years, DASCs featuring asymmetric coordination have attracted considerable research interest owing to their distinctive structural and electronic properties. A brief summary of the historical evolution and latest progress of ADASCs is presented in Fig. 2 [41–48]. ADASCs specifically refer to a class of catalysts that employ precise structural design strategies to regulate metal atom types or coordination microenvironments. This enables dual-metal active sites anchored on a specific support to form a diatomic site catalytic system exhibiting dual asymmetry in both geometric configuration and electronic state. The asymmetric configuration of ADASCs mainly stems from the asymmetry of the heteronuclear metal center, that of coordination environments, and their combination. Currently, the primary strategies to break the structural symmetry of dual-atom catalysts include: (1) Doping different heteroatoms or engineering unsaturated heteroatomic coordination to effectively break the symmetry of coordination environments and generate heterogeneous active sites. (2) Incorporating heteronuclear metals with significant differences in electronegativity [49–53], atomic radius, or *d*-electron configuration to construct asymmetric coordination environments, enabling precise regulation of geometric structures while optimizing reaction kinetics and mechanisms. This unique structure–function relationship offers novel insights for overcoming the kinetic bottleneck of slow multi-electron transfer reactions. Leveraging this intrinsic asymmetric structure, ADASCs exhibit significantly superior ORR/OER performance compared to SASCs and symmetric dual-atom site catalysts (SDASCs). The synergistic mechanisms enhancing their catalytic activity and stability primarily include: (1) Establishing a directed electron transfer system that precisely regulates the electronic states of active sites to meet the requirements of oxygen electrocatalysis [54]. The asymmetric coordination structure of ADASCs disrupts the uniform distribution of the active site electron cloud, forming a spontaneously oriented electron transfer pathway that enables bidirectional regulation of the *d*-band center (*E*_d_). During the ORR process, moderately lowering *E*_d_ weakens the adsorption strength of the oxygen intermediates [55], reduces the reaction energy barrier for the protonation step, and accelerates reaction kinetics. During the OER, moderately increasing *E*_d_ enhances the adsorption and activation efficiency of the ^*^OH intermediate, providing a thermodynamic advantage for subsequent elementary reactions (e.g., ^*^OH → ^*^O). Tang et al. synthesized an asymmetrically coordinated dual-site catalyst FeCo-N_3_O_3_@C, where Fe and Co atoms are coordinated to N and O atoms, respectively, and linked via bridging N/O atoms [56]. Experimental characterization and theoretical calculations demonstrate that the Fe–N_3_ and Co–O_3_ moieties serve as dedicated active sites for the ORR and OER, respectively, facilitating the 4e^−^ reduction pathway. The enhanced bifunctional activity originates from highly electronegative N atoms lowering the Fe potential energy (thereby weakening ^*^O adsorption) and O atom coordination raises the Co potential energy (thus enhancing ^*^OOH formation). (2) Differentiated coordination and dual-center synergy optimize the adsorption strength and reaction pathways of oxygen intermediates [57]. For instance, in the ORR, one active site selectively adsorbs and stabilize oxygen intermediates, while the adjacent site weakens the O–O bond via electron transfer, thereby synergistically lowering the O_2_ dissociation energy barrier. Furthermore, this structural feature favors the adoption of the O_2_ dissociation reaction pathway, which not only accelerates reaction kinetics but also avoids the formation of the by-product H_2_O_2_, thus enhancing catalytic stability. In the OER, the desorption of ^*^OH and its coupling with O can be separately regulated, preventing excessive or insufficient adsorption of a single intermediate at symmetric sites, which optimizes the overall reaction pathway. Yuan et al. synthesized Fe–Co DACs with heteronuclear metal centers via molecular chelation and ion coupling strategies [58]. Co serves as the primary catalytic center for the 4e^−^ process, while Fe sites predominantly adsorb ^*^OH. This functional division reduces the theoretical overpotential for the acidic oxygen reduction reaction from 1.14 to 0.43 V, optimizes the adsorption strength of oxygen intermediates, and enhances the selectivity of the 4e^−^ pathway. Although ADASCs exhibit exceptional performance in oxygen electrocatalysis, research in this field remains in its nascent stage. Specifically, precise regulation of the individual coordination environments of two (identical or distinct) metal atoms in dual-atom architectures remains challenging—a limitation that also obscures the reaction mechanisms. Additionally, doping engineering and analogous techniques frequently induce randomly distributed defects, making it elusive to simultaneously achieve precisely defined, effective asymmetric configurations and structural homogeneity. Concomitantly, asymmetric structures (e.g., unsaturated coordination) can trigger active center instability under reaction conditions, leading to atomic migration or agglomeration that compromises catalytic durability. Consequently, designing effective strategies for constructing uniform asymmetric dual-atom structures requires establishing a balance between symmetry breaking and structural stability to attain and sustain optimal catalytic performance.

**Fig. 3:**
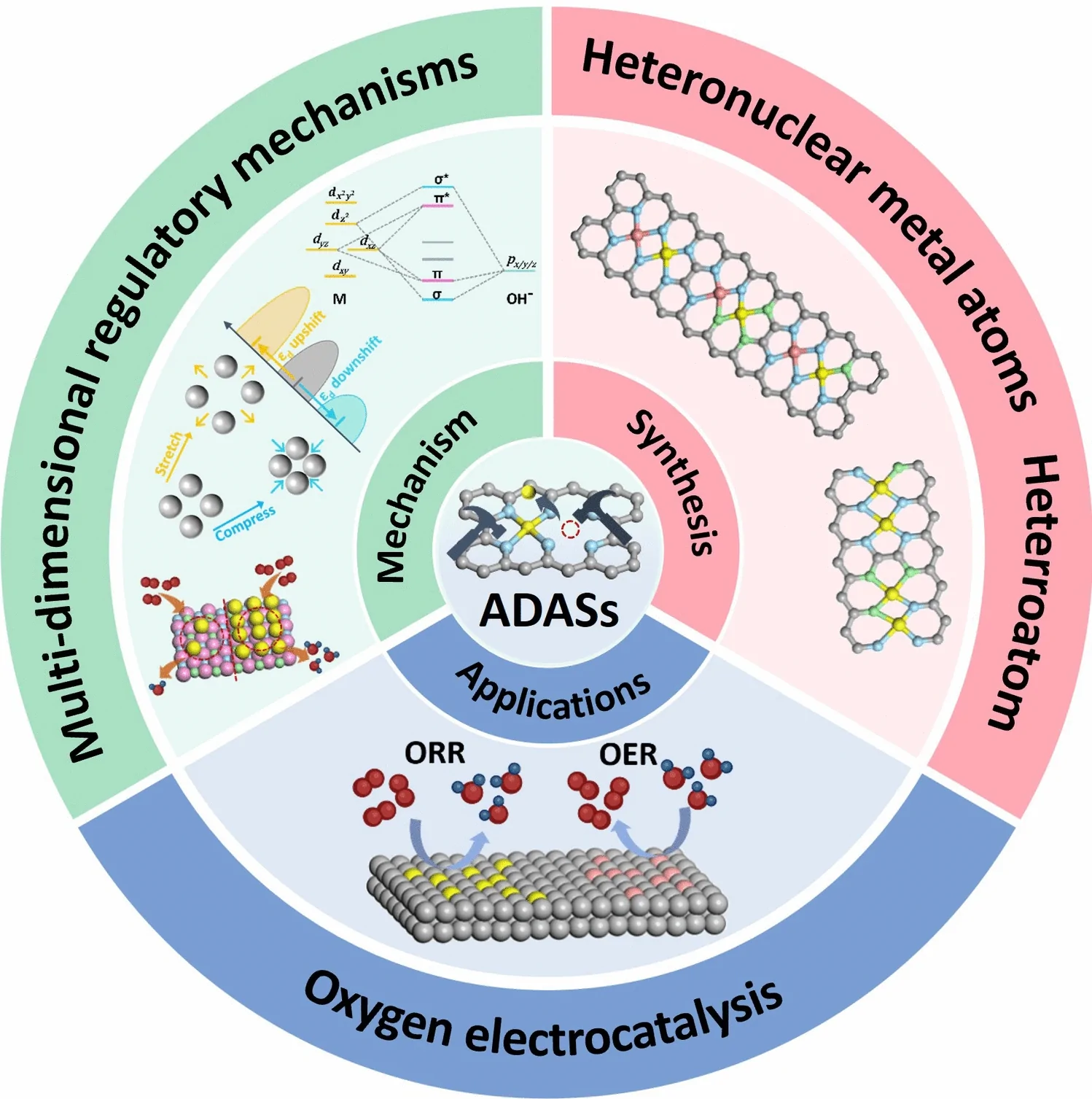
Overview of multi-dimensional performance optimization mechanisms, strategies for constructing asymmetric structures, and applications of ADASCs

Hence, this review systematically summarizes the potential multi-dimensional performance optimization mechanisms of ADASCs, including enhancing active site density (SD), optimizing anchoring effects, modulating *E*_d_, tailoring spin structures, promoting orbital hybridization, matching energy level, and regulating charge states and electrical conductivity. These insights lay a foundation for understanding the effective construction of ADASCs. Building on this mechanistic understanding, we further summarize the diverse methods for constructing ADASCs, with a focus on heteronuclear metal centers and heterogeneous nonmetallic heteroatom doping engineering, which enable precise modulation of electronic structures and coordination environments in ADASCs. Systematic research not only facilitates the precise construction of ADASCs but also can be used to effectively enhance oxygen electrocatalysis performance, thereby revealing the structure–performance relationships between asymmetric dual-atom configurations and catalytic properties (Fig. 3). Finally, the existing challenges and emerging opportunities faced by ADASCs in electrochemical energy conversion and storage research are systematically elaborated.

## Multi-dimensional Performance Optimization Mechanisms

Compared to SASCs, symmetric dual-atom site catalysts (SDASCs) not only retain the inherent advantages of SASCs but also afford increased active SD to further boost catalytic activity. Nevertheless, their homonuclear metal centers and uniform coordination electronic environments render them poorly suited to meet the adsorption requirements of diverse intermediates involved in multi-step reactions [31, 59, 60]. Against this backdrop, ADASCs, relying on the dual asymmetry of either heteronuclear metal centers or homonuclear metal centers with distinct coordination environments, have become the key to addressing the aforementioned limitations. To gain an in-depth understanding of the underlying mechanisms the catalytic reaction optimization by ADASCs, and to enable targeted, precise construction of ADASCs with specific structures, the advantages and limitations of optimization mechanisms are discussed in detail from the following six aspects: enhancing active SD, optimizing anchoring effects, regulating *E*_d_, optimizing spin states, promoting orbital hybridization and matching energy levels, and regulating charge states and electrical conductivity.

### Enhancing Density of Active Sites

**Fig. 4:**
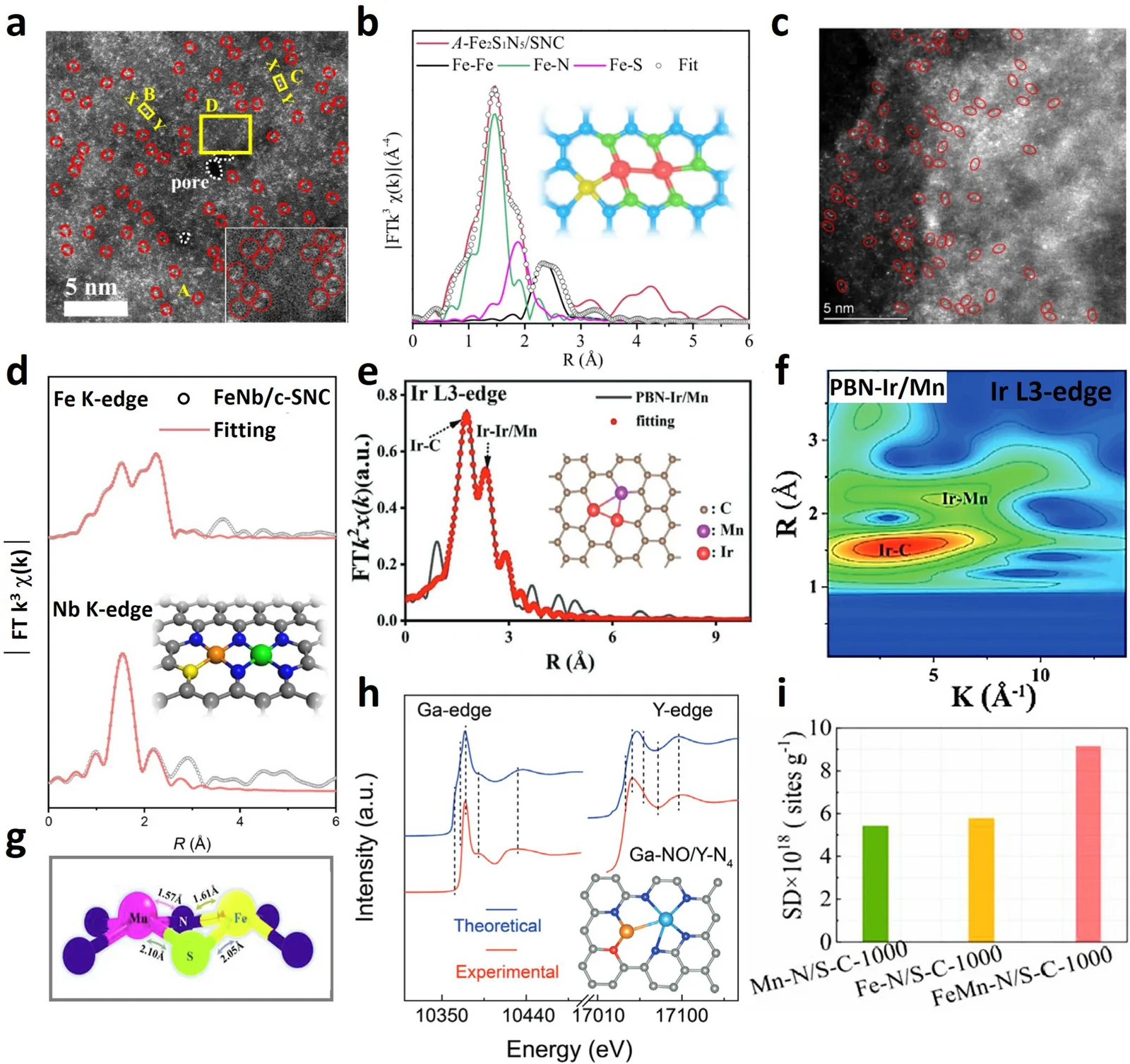
ADASCs optimize the density of active sites. **a** AC-HAADF-STEM plots and **b** structure characterizations of *A-*Fe_2_S_1_N_5_/SNC. Reproduced with permission [46]. Copyright 2024, Springer Nature. **c** AC-HAADF-STEM image and **d** EXAFS spectrum fitting of FeNb/c-SNC. Reproduced with permission [64]. Copyright 2024, American Chemical Society. **e** EXAFS fitting curves and **f** WT-EXAFS plots of PBN-Ir/Mn at Ir L3-edge. Reproduced with permission [66]. Copyright 2025, Wiley-VCH. **g** Optimized model of FeMn-N_5_/S–C. **h** Structure characterizations of Ga/Y-CNP. Reproduced with permission [69]. Copyright 2025, Wiley-VCH. **i** Comparison of SD. Reproduced with permission [70]. Copyright 2024, Elsevier

Similar to SASCs, the active sites of ADASCs are atomically dispersed. Theoretically, every available anchoring site on the support surface (e.g., specific coordination vacancies in nitrogen-doped carbon materials) can be utilized, providing a pathway to achieve high-density active sites. For instance, Zhang et al. designed a highly defective S, N-co-doped carbon support using a multilayer stabilization strategy (involving defect trapping and coordination anchoring) to anchor asymmetric dual-atom sites [46]. The resulting catalyst (A-Fe_2_S_1_N_5_/SNC) features densely dispersed asymmetric Fe_2_ sites with a FeS_1_N_2_–FeN_3_ configuration (Fig. 4a, b). It exhibits a high metal loading of 6.72 wt% and demonstrates significantly superior activity compared to low-loading SASCs. Additionally, A-Fe_2_S_1_N_5_/SNC exhibits an ultra-low overpotential during the OER process (*η*_10_ = 193 mV), maintaining continuous operation for over 2000 h.

High active SD is not a mere "quantitative increase"; rather, when integrated with the electronic heterogeneity and synergistic effects of dual sites, it imparts multi-dimensional impacts on the catalytic system. The value of high active SD enabled by ADASCs should be considered in conjunction with the "dual-site synergistic characteristics" of ADASCs, rather than viewing "quantity" in isolation. Its advantages are primarily manifested in three aspects:

1. Markedly boosting the catalytic reaction rate and mass activity [61–63]. In low-concentration reactant systems, high SD elevates the collision frequency between reactants and active centers, avoiding " active site inactivity caused by insufficient reactants." For multi-step sequential reactions, it shortens the "migration distance of intermediates across distinct sites," reduces mass transfer resistance, and mitigates intermediate desorption and loss. For instance, Zhao et al. synthesized asymmetric Fe-Nb dual-atom catalysts (Fe-Nb/c-SNC) via pyrolysis [64]. X-ray absorption spectroscopy (XAS) and density functional theory (DFT) calculations confirmed that the catalysts possess an asymmetric coordination configuration of Fe–S_1_N_3_ and Nb–N_4_ (Fig. 4c, d). The loadings of Fe and Nb are 1.85 wt% and 1.83 wt%, respectively, and the total metal loading is higher than that of Fe/c SASCs and Nb/c SASCs. Electrochemical analysis revealed that Fe-Nb/c-SNC exhibits the highest turnover frequency (TOF) (33 s^−1^) and SD (27 × 10^19^ sites g^−1^). Benefiting from the highest SD and intrinsic activity, the collision frequency between the Fe-Nb dual-atom sites and the reactant O_2_ is significantly increased, which improves the reaction rate. Additionally, the strong interaction between the dual-atom sites optimizes the desorption energy of the key intermediate (^*^OH), making the adsorption energy of Fe–^*^OH close to the apex of the volcano plot. As a result, the catalyst exhibits superior ORR performance and stability.
2. Maximizing atomic utilization efficiency and substantially cutting noble metal costs [65]. High SD enables reduced noble metal loading while "meeting activity requirements"; it also offsets the limitation of "lower intrinsic activity per single site compared to noble metals," achieving performance surpassing that of SASCs via "quantitative advantage." Liu et al. incorporated Mn into a noble metal-based Ir catalyst using atomic layer deposition technology, successfully synthesizing a two-dimensional bimetallic catalyst denoted as PBN-Ir/Mn (Fig. 4e, f) [66]. This catalyst uses significantly less noble metal Ir compared to PBN-Ir and similar Ir SASCs. Specifically, the Ir loading is 0.47 μg cm^−2^ (0.24 wt%), which is remarkably lower than that of PBN-Ir (0.74 wt%) and other recently reported single-atom Ir-based bifunctional catalysts. Meanwhile, PBN-Ir/Mn exhibits lower overpotentials and superior activity in both the HER and OER compared to commercial counterparts (Pt/C for HER and IrO_2_ for OER), achieving a comprehensive leap in water splitting performance with an ultra-low noble metal dosage. Additionally, PBN-Ir/Mn exhibits an ultra-low overpotential during the OER process (*η*_10_ = 220 mV), maintaining continuous operation for over 100 h.
3. Enhancing the "spatial efficacy" of dual-site synergy [67, 68]. The synergy of ADASCs depends on the "spatial proximity of dual-atom sites." High SD ensures the "dense distribution of synergistic units (M_1_–M_2_)," avoiding "low synergistic efficiency caused by sparse synergistic sites." Han et al. designed and synthesized a high-density Ga-Y dual-atom catalyst (Ga/Y-CNP), in which Ga–Y diatomic pairs are anchored on a N-P co-doped carbon-based support [69]. The loadings of Ga and Y reach as high as 14.1 wt%, which enables the dense distribution of Ga–Y dual-atom pairs, ensures the spatial accessibility of dual-atom sites, and improves synergy efficiency (Fig. 4h). Further verification via XAS and DFT calculations shows that the two atoms mutually modulate electronic states through spatial proximity. Ga sites mainly promote the reduction of CO_2_ to ^*^CO, while Y sites preferentially catalyze the reduction of NO_3_^−^ to hydroxylamine (^*^NH_2_OH). Subsequently, ^*^CO and ^*^NH_2_OH are spontaneously coupled at the Ga–Y sites to form C–N bonds, resulting in a urea yield of 41.9 mmol h^−1^ g^−1^ and a Faradaic efficiency of 22.1%, which are far superior to those of single-atom and nanoparticle catalysts, maintaining continuous operation for over 120 h. Tan et al. successfully prepared the dual-atom FeMn–N/S–C catalyst by adopting a simple micelle template strategy and a modified polymer pyrolysis route [70]. The in situ nitrite reduction method confirmed that the active SD of this dual-atom catalyst reaches 9.169 × 10^18^ sites g^−1^, which is much higher than that of single-atom Mn–N/S–C (5.423 × 10^18^ sites g^−1^) and Fe–N/S–C (5.782 × 10^18^ sites g^−1^). Its high SD enables the dense distribution of Fe–Mn synergistic units (Fig. 4g, i). As a result, with the help of the abundant active sites formed by the N, S-co-doped structure and the synergistic effect of Fe and Mn dual atoms, the activity and stability of the catalyst is significantly improved compared with that of SASCs.

However, high active SD is not a case of "the higher the better"; active site densification also gives rise to issues such as decreased structural stability, imbalanced selectivity, and impaired mass transfer. These problems are detailed as follows: (1) Elevated risk of site aggregation, leading to reduced catalyst stability [71, 72]. High active SD implies narrowed metal atomic spacing—for ADASCs, the dual-atom spacing is typically 0.2–0.3 nm, and the spacing between adjacent dual sites can be < 1 nm under high-density conditions. During reactions at high temperatures or high electrolyte concentrations, sites tend to migrate and aggregate; excessive increases in SD may cause unsaturated coordination of some dual-atom sites, which weakens the metal–support binding force and leads to easy desorption and loss of atoms. (2) Intensified "competitive adsorption" between sites, resulting in decreased reaction selectivity [62]. Under high active SD, the distance between adjacent sites is excessively small (< 1 nm), which easily triggers competitive adsorption of intermediates or activation of side reaction pathways, thereby reducing the yield of target products. (3) Augmented mass transfer resistance, limiting the enhancement of reaction rate [61]. High active SD generally requires catalysts to have a high specific surface area. However, when the metal loading approaches the saturation capacity of the support, this tends to cause blockage of support pores. Such blockage prevents reactants/products from rapidly diffusing to internal active sites and also hinders the diffusion of electrolytes/reactants between sites, leading to intensified concentration polarization and increased voltage loss under high current density. Therefore, although high active SD is crucial for ADASCs to overcome the performance bottleneck of SASCs, it is necessary to strike a balance between "density enhancement" and "maintaining stability, selectivity, and mass transfer performance."

### Optimizing Anchoring Effects

For atomically dispersed catalysts, the core challenge lies in suppressing the migration and agglomeration of metal atoms under reaction conditions. Traditional SASCs rely on the coordination of metal atoms with four nitrogen atoms to form a highly symmetric M–N_4_ geometric configuration. This single M–N_4_ anchoring configuration not only results in a uniform electronic structure of SASCs, making it difficult to optimize the binding strength with reaction intermediates, but also limits further stabilization due to its rigid structure. Moreover, driven by thermodynamics, metal atoms tend to detach from the anchoring sites, leading to irreversible deactivation of the catalyst. Therefore, precisely designing the local atomic configuration to break symmetry and constructing stronger metal–support interactions have become core strategies to enhance the intrinsic stability of catalysts [20, 73–75]. ADASCs are developed based on this concept; by constructing an anchoring structure with heteronuclear metal synergy, they provide a new pathway to simultaneously achieve high stability and high activity.

The core of the anchoring effect lies in the formation of stable bonding between the support and dual-atom active sites via chemical interactions (e.g., coordination bonds, electrostatic interactions), which "immobilizes" the dual-atom sites on the support surface or within pores. Essentially, it serves as a "spatial and chemical stability regulation mechanism," with specific manifestations in the following three aspects:

1. Achieving "site-specific anchoring" of dual-atom sites through coordination bonds. This coordination-driven anchoring constitutes the dominant form of the anchoring effect in ADASCs: The support forms coordination bonds with dual atoms via specific heteroatoms (e.g., N, O, S, P), thereby "locking" the dual-atom sites in fixed spatial locations. Additionally, the strength of the anchoring effect can be modulated by the coordination bond energy. Mo et al. innovatively introduced bimetallic sites and weakly electronegative S atoms into M–N–C catalytic materials, successfully synthesizing Co–Fe dual-atom sites on S, N-co-doped carbon as a highly efficient ORR electrocatalyst (denoted as Co–Fe-SNC) [76]. Specifically, S doping optimizes the bond energy, weakens the adsorption strength of ^*^OH, and reduces the ORR reaction energy barrier. The effectively overcomes the limitations of traditional single heteroatoms, enables the synergistic enhancement of intermediate adsorption and activation by S and N, and thus significantly boosts ORR performance.
2. Inhibition the aggregation and migration of dual-atom sites. The core function of the anchoring effect in ADASCs is to address the "propensity of dual atoms to aggregate" through steric hindrance or chemical binding constraints. The pore structure (e.g., mesopores, micropores) or surface defect sites of the support can form "spatial confinement" for dual atoms. For instance, Liu et al. constructed a defect-engineered FeMn dual-atom catalyst (FeMnDSA/dNC) using porous N-doped carbon as the support and achieved the anchoring effect via a customized trinuclear defect trapping strategy [77]. Specifically, the porous structure of the support forms a physical "spatial confinement," while its surface defect sites provide chemical constraints via coordination interactions. These two aspects synergistically inhibit the aggregation and migration of FeMn dual atoms, stabilizing the dispersed state of the dual-atom pairs. This anchoring effect also optimizes the electronic structure, promoting the hybridization between Fe 3*d* orbitals and O 2*p* orbitals. As a result, the catalyst exhibits a half-wave potential (*E*_1/2_) of 0.921 V, and the zinc–air battery assembled with it maintains stable cycling for over 500 h. Meanwhile, the rigid framework on the support surface (e.g., the graphite-like structure of MOF-derived carbon) can impede the lateral diffusion of dual atoms, precluding the formation of multi-atom clusters (> 2 atoms). The coordination bonds generated by the anchoring effect provide "chemical restraining forces" for dual atoms, elevating their migration energy barrier. Owing to the high nitrogen content and well-defined microporous structure of ZIF-8, Ao et al. employed it as a confinement scaffold and adopted a host–guest confinement strategy to fabricate the FeCu–NC heteronuclear bimetallic single-atom catalyst [78]. EXAFS characterization confirmed the formation of Fe–N_4_ and Cu–N_4_ configurations, with an interatomic distance of 2.3–2.4 Å between the dual atoms. Fe and Cu are anchored via Fe–N and Cu–N coordination bonds, which provide chemical binding forces that significantly increase the migration energy barrier of the dual atoms and enhance the anchoring effect. Meanwhile, the proton exchange membrane fuel cells (PEMFCs) assembled with this catalyst retained 93% of the catalyst’s initial performance after 5000 cycles, demonstrating excellent stability.
3. Stabilizing the "asymmetric structure" of dual-atom sites. The core advantage of ADASCs hinges on the "chemical environment discrepancy between the two metal atoms" (i.e., asymmetry), and the anchoring effect can sustain this asymmetry by modulating the coordination environment. Chen et al. prepared a Cu-In DAC via high-temperature pyrolysis, using ZIF-8 derived nitrogen-doped carbon as the support [79]. EXAFS fitting confirmed that Cu is anchored in a Cu–N_4_ configuration (coordinated only with N), while In is anchored in a In–N_4_O_2_ configuration (coordinated with both N and O). This difference in coordinating atoms creates an asymmetric chemical environment, and the anchoring effect maintains this asymmetry by precisely regulating the coordination bonds between N/O and the dual atoms. The asymmetry enables In to stabilize the ^*^ON intermediate and Cu to directionally adsorb ^*^CO, leading to the formation of the key intermediate ^*^ONCO and thus promoting urea electrosynthesis. Furthermore, the anchoring effect can also avert "metal–metal bond cleavage" of dual atoms during reactions (e.g., bond dissociation induced by high potential in acidic OER) [80, 81], preserving the integrity of the dual-atom unit.

In summary, the anchoring effect serves as a "structural stabilizer" for ADASCs. It maintains the stability and asymmetry of dual-atom sites through chemical coordination and spatial confinement, with its core function dedicated to enhancing catalyst durability. However, constrained by factors such as support performance, synthesis processes, and reaction conditions, the exertion of its role has significant limitations: Either the support fails to provide sufficient and precise anchoring sites, or there is a trade-off between anchoring strength and catalytic activity, or it is difficult to withstand extreme reaction environments. These limitations may lead to insufficient anchoring strength, uneven site distribution, and even indirect restrictions on catalytic activity and selectivity, thus necessitating the development of targeted solutions.

### Regulating d-Band Centers

The *E*_d_ is a key electronic structure descriptor that characterizes the energy state distribution of *d*-electrons in transition metals. Its position relative to the Fermi level directly determines the adsorption strength of reaction intermediates on the catalyst surface [82, 83]. According to *E*_d_ theory, ideal catalytic performance requires moderate adsorption energy for key reaction intermediates [84]. Specifically, excessively weak adsorption increases the reaction overpotential, while excessively strong adsorption poisons the active sites—these two scenarios both deteriorate catalytic performance. Therefore, precise modulation of *E*_d_ is a central strategy for optimizing the intrinsic activity and selectivity of catalysts.

**Fig. 5:**
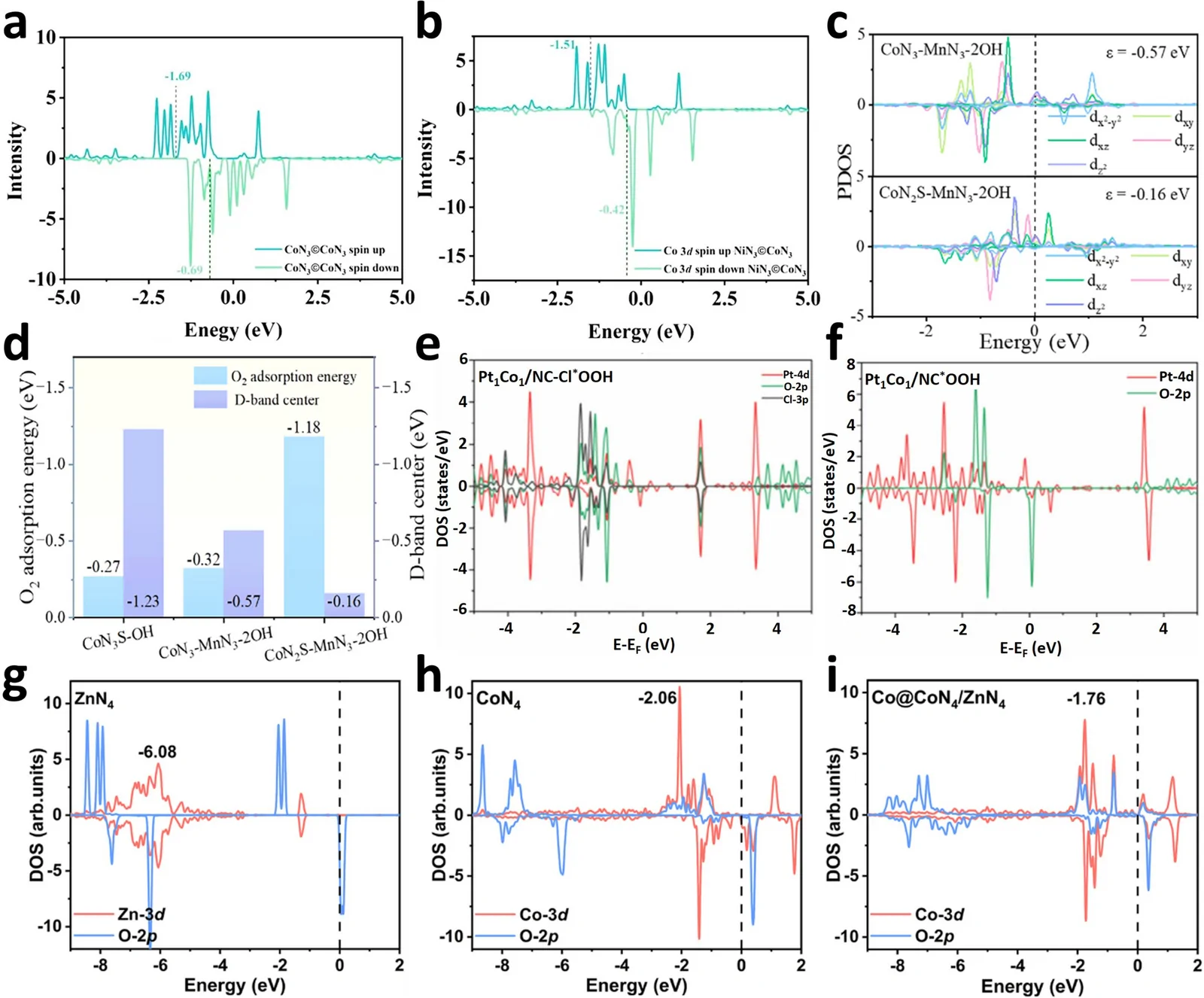
ADASCs optimize the *d*-band center. **a** PDOS of CoN_3_@CoN_3_-NC and **b** NiN_3_@CoN_3_-NC. Reproduced with permission [88]. Copyright 2023, Elsevier. **c** PDOS of CoN_2_S-MnN_3_-2OH and CoN_3_-MnN_3_-2OH. **d** Comparison of the O_2_ adsorption energy and E_d_. Reproduced with permission [47]. Copyright 2025, Wiley-VCH. **e** PDOS analysis of Pt_1_Co_1_/NC–Cl and **f** Pt_1_Co_1_/NC after adsorption of ^*^OOH. Reproduced with permission [91]. Copyright 2025, Wiley-VCH. PDOS of **g** ZnN_4_, **h** CoN_4_, and **i** Co@CoN_4_/ZnN_4_ models. Reproduced with permission [92]. Copyright 2022, Elsevier

The regulation of *E*_d_ is primarily achieved through two interrelated electronic structure parameters: the *d*-electron population and the *d*-band width [85–87]. In contrast to SASCs and SDASCs, ADASCs demonstrate unique advantages owing to synergistic effects between their heteronuclear metal centers: SASCs offer only a single regulatory dimension—relying on the support ligand field and lacking the ability to synergistically optimize adsorption for multiple intermediates—while SDASCs exhibit unidirectional regulation due to their homonuclear centers. On one hand, by incorporating heterometallic elements with distinct *d*-electron configurations, ADASCs enable direct and synergistic modulation of the local *d*-electron population at the active sites, fundamentally altering their electron donation/acceptance properties and providing a foundation for preliminary adsorption energy optimization. For instance, Zhang et al. constructed a Ni-Co bimetallic atom catalyst embedded in N-doped graphitized carbon, with a structural model featuring a covalently coupled Ni–Co atomic pair [88]. DFT calculations of the projected density of states (PDOS) show that the incorporation of Co into the NiN_3_ framework leads to an asymmetric distribution of the PDOS of Ni 3*d* orbitals, indicating an interaction between the NiN_3_ and CoN_3_ active sites. In addition, the *E*_d_ of the Co 3*d* orbitals in the NiN_3_–CoN_3_–NC structure is closer to the Fermi level (1.51 eV), suggesting stronger adsorption of key intermediates at this active site (Fig. 5a, b). This synergistic effect promotes the formation of the key ^*^COOH intermediate, improving reaction activity and stability. Moreover, ADASCs can construct a more complex coordination environment with the support (e.g., different coordination numbers and types of coordinating atoms), and further adjust the energy level position of the *E*_d_ through the ligand effect [89]. Zhao et al. synthesized a novel asymmetrically coordinated Co-Mn dual-atom catalyst via an adsorption–pyrolysis process using a bimetallic imidazole framework [47]. The catalyst consists of adjacent S/N dual-coordinated Co atoms and N-coordinated Mn atoms (CoN_2_S-MnN_3_), anchored on N-doped carbon. DFT calculations of the PDOS show that the *E*_d_ of CoN_2_S-MnN_3_–2OH is higher than that of CoN_3_S–OH (−1.23 eV), meaning CoN_2_S-MnN_3_–2OH exhibits more favorable adsorption energy for ORR intermediates. The CoN_2_S-MnN_3_–2OH configuration shows a larger overlap region between the active sites and the molecular orbitals of O_2_, which is directly related to stronger O_2_ adsorption (Fig. 5c, d). This enhanced O_2_ adsorption is attributed to the increased hybridization between the d orbitals of Co and Mn and the antibonding *π* orbitals of O_2_, which promotes electron transfer and optimizes the binding energy of ORR intermediates. Therefore, CoMn-NSC catalyst exhibits ORR activity with a high *E*_1/2_ of 0.901 V, and the assembled ZABs has ultra-long life spans of up to 1000 h.

On the other hand, the combination of two metals with different atomic radii and electronegativities can introduce a unique strain effect (compressive or tensile) through lattice mismatch, altering the metal–metal bond length. This bond length change, in turn, directly affects the degree of d orbital overlap and the *d*-band width [90]. Zhang et al. prepared a Pt–Co dual-atom site catalyst with an asymmetrically coordinated Pt(Cl)–Co–N_6_ structure featuring axial Cl ligands [91]. Among the two heteronuclear metal atoms, Pt has a higher electronegativity (2.28) than Co (1.88), and the differences in their atomic radii and electronegativities induce lattice mismatch. AC-HAADF-STEM characterization confirmed that the Pt–Co bond length is 2.94 Å, which is slightly longer than that of the symmetric PtCo dual-atom catalyst (theoretical bond length of ~ 2.85 Å), resulting in a tensile strain effect. Combining XAFS and DFT calculations, this change in bond length directly modifies the degree of overlap between Pt 4*d* and Co 3*d* orbitals, narrowing the *d*-band width (Fig. 5e, f). Meanwhile, the electronegativity difference drives electron transfer from Co to Pt, synergistically optimizing the *E*_d_ and enhancing the ^*^OOH adsorption capacity. Ultimately, the *E*_1/2_ of the ORR in acidic media reaches 0.841 V. After 5000 catalytic cycling via CV measurement, the *E*_1/2_ decreases by 12 mV, indicating the stability of the asymmetric structure in acidic ORR. Zheng et al. used MOF materials as precursors and synthesized a carbon-based catalyst containing Zn/Co bimetallic atoms via a cavity confinement and post-adsorption strategy [92]. XANES and EXAFS spectra confirm that Zn atoms transfer electrons to Co atoms through graphite-conjugated *π* bonds, thereby further optimizing the electronic configuration of Co central atoms. DFT calculations indicate that the electron transfer effect between the bimetallic components optimizes the electronic configuration of CoN_4_ active sites, weakens the adsorption strength with O_2_ molecules, and accelerates the formation of the key ^*^OOH intermediate. Consequently, the ZnCo-NC-II catalyst exhibits ORR catalytic activity comparable to that of commercial Pt/C under both acidic and alkaline conditions, and by CV measurement, after 10,000 accelerated durability cycles, its *E*_1/2_ decreases by 3 mV, indicating the stability of the asymmetric structure (Fig. 5g, h). Therefore, ADASCs possess multiple mechanisms—including strain effect, ligand effect, and electron transfer—which enable them to achieve synergistic and bidirectional regulation (upward or downward shift) of *d*-electron population and d-band width. In this way, the optimal adsorption strength of key reaction intermediates is realized, and the overall catalytic performance is enhanced.

However, *E*_d_ is influenced by the synergistic coupling effects of *d*-electron distribution, strain effects, and ligand effects, making it difficult to establish quantitative structure–activity relationships and challenging to achieve targeted regulation. Furthermore, surface reconstruction and intermediate adsorption during catalysis cause real-time fluctuations in E_d_, which cannot be precisely tracked at the atomic scale by current characterization techniques. Therefore, the integration of DFT + high-throughput screening to predict the impact of multi-mechanism coupling on E_d_, characterization techniques such as PDOS and XAFS for quantitative model construction, and in situ tracking of dynamic changes in *E*_d_ and electronic structure during reactions, in real time, constitutes a key strategy to overcome the bottleneck of precise *E*_d_ regulation in ADASCs and enhance their catalytic performance and application potential.

### Optimizing Spin Configurations

In electrocatalytic reactions, the spin configuration is a fundamental electronic property that critically determines catalytic efficiency [3, 93, 94]. On one hand, the degree of spin matching between the reactant molecule (e.g., ground-state O_2_ is a triplet with two parallel spin electrons) and the catalyst's active sites directly influences the activation energy barrier [95]. According to the Sabatier principle [96], excessively weak binding fails to activate reactants, while overly strong binding impedes product desorption; consequently, an optimal binding energy is essential for maximizing the reaction rate. Specifically, when the spin state of the catalyst's metal center matches that of the reactant molecule, orbital hybridization and electron transfer become most effective—this synergy ensures the binding energy falls within the optimal range dictated by the Sabatier principle. On the other hand, the spin polarization effect can steer the electron transfer pathway and lower the reaction energy barrier [97], For instance, in the ORR, spin-polarized electrons can preferentially occupy the antibonding orbitals of the ^*^OOH intermediate, facilitating its dissociation. Furthermore, the spin state governs reaction selectivity. For example, in the CO_2_RR, low-spin Co^2+^ tends to produce CO, whereas high-spin Co^2+^ favors C–C coupling to generate C_2_ products [98, 99], this difference arises from the distinct electron cloud distributions of low-spin and high-spin Co^2+^, which modulate the adsorption strength of key intermediates (e.g., ^*^CO) and thus steer the reaction toward different product pathways.

**Fig. 6:**
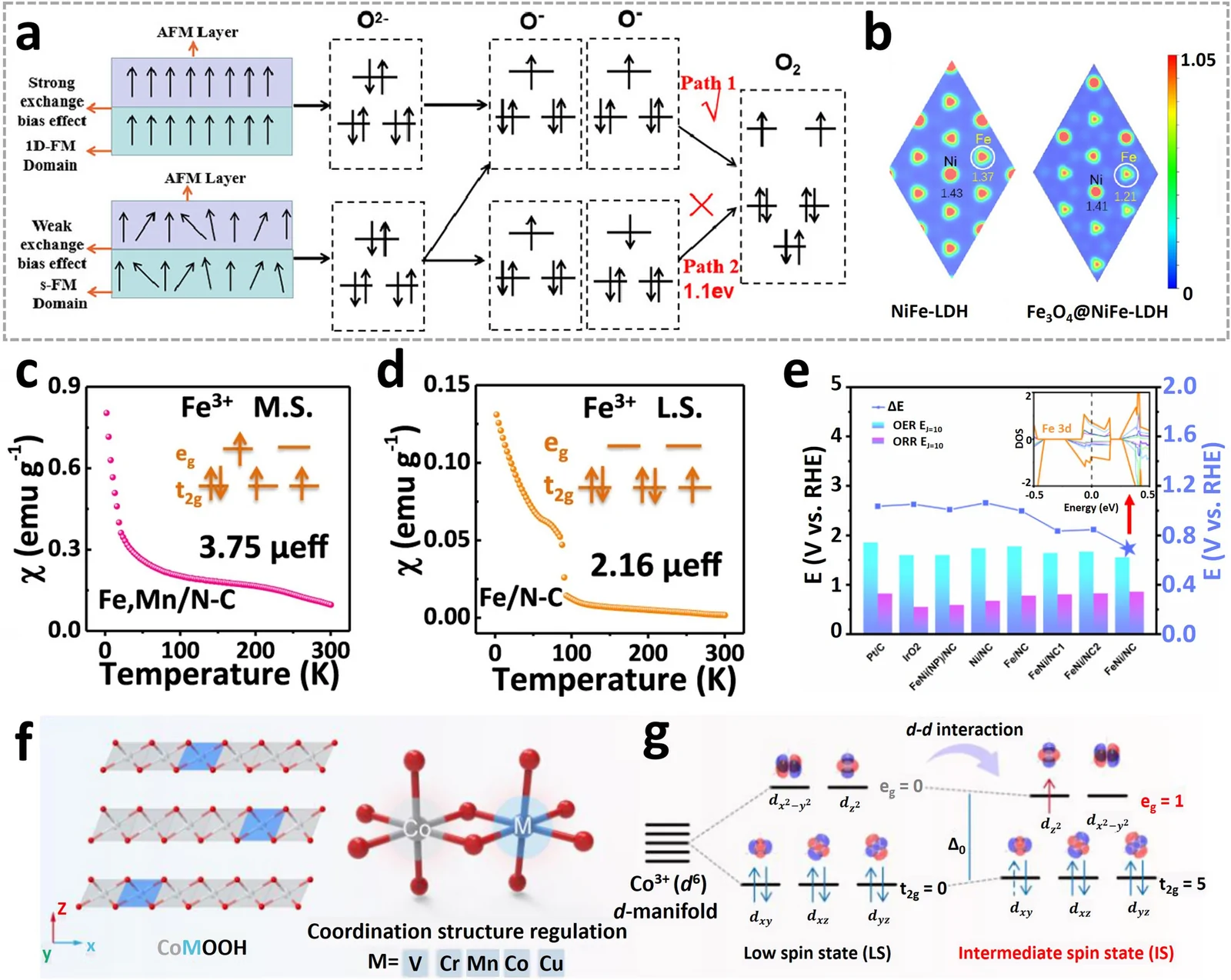
ADASCs optimize the spin state. **a** Exchange bias effect. **b** Electron density of NiFe-LDH and Fe_3_O_4_@NiFe-LDH. Reproduced with permission [103]. Copyright 2024, American Chemical Society. Magnetic susceptibility of **c** Fe, Mn/N–C and **d** Fe/N–C. Reproduced with permission [48]. Copyright 2021, Springer Nature. **e** Comparison of performance and DOS for Fe/Ni–N-C. Reproduced with permission [104]. Copyright 2021, Elsevier. **f** Coordination structure regulation and **g** the spin state regulation of CoOOH. Reproduced with permission [105]. Copyright 2024, Wiley-VCH

There is no strict spatial constraint on the separation between the two atoms at different active sites in ADASCs, making it difficult to form close-proximity bimetallic configurations. However, due to the long-range electronic interactions between metal centers, ADASCs still exhibit significantly superior performance to SASCs in electrochemical energy conversion reactions [100]. According to the Goodenough–Kanamori rule (an important rule in solid-state physics and materials science used to describe the relationship between exchange interactions (QSEI) and magnetism in transition metal compounds [101, 102].), spin coupling can occur between two adjacent magnetic metal atoms through superexchange or double exchange mechanisms, leading to tunable changes in the effective magnetic moment and spin state of the metal active sites. In the heteronuclear metal active site configuration of ADASCs, the two heterometals differ in intrinsic *d*-electron count and Hund’s rule stable states; this difference often breaks the inherent high-/low-spin state of a single metal center, forming a synergistic spin configuration with lower energy. This configuration is more conducive to spin-matched electron transfer with reactants, thereby significantly reducing the reaction energy barrier and improving catalytic efficiency in target electrochemical reactions (e.g., ORR, OER). For instance, Cao et al. regulated the spin polarization of NiFe layered double hydroxide (NiFe-LDH) by establishing an internal magnetic field via a built-in magnetic core; this induced an exchange bias effect at the interface between the core and the NiFe-LDH shell. Specifically, this interface acts as a "spin filter" that selectively removes electrons with magnetic moments opposite to those of the magnetic core [103]. Spectroscopic and magnetic characterizations revealed spin coupling at the heteronuclear Ni–Fe sites: The magnetic moment of Fe^3^⁺ decreased from 4.5 to 3.6 μ_B_. electron paramagnetic resonance (EPR) and XANES analyses confirmed a transition of the Fe^3^⁺ sites from a high-spin to a low-spin state. The superexchange interaction between Ni and Fe—mediated by oxygen bridges—induced reconstruction of the Fe 3*d* and O 2*p* orbital hybridization, which reduced the adsorption energy of the ^*^O intermediate and broke the limitation of a single spin state (Fig. 6a, b). This synergistic spin configuration optimized the spin matching with O_2_, endowing the catalyst with outstanding electrocatalytic activity for the OER, with a low overpotential of 196 mV at a current density of 30 mA cm^−2^ and displays outstanding durability, where the current retention rate remains at 86.4% even after the 24 h cycle test. Yang et al. further found that introducing atomically dispersed Mn–N moieties into the Fe–N–C catalyst triggers spin coupling between Fe^3+^ (*d*^*5*^) and Mn^3+^ (*d*^*4*^) via the superexchange mechanism—driven by differences in *d*-electron count and Hund's rule stable states [48]. Mössbauer spectroscopy results show that Fe^3+^ transitions from a low-spin state (*t*_*2g*_^*5*^*e*_*g*_^*0*^) in Fe/N–C to an intermediate-spin state (*t*_*2g*_^*4*^*e*_*g*_^*1*^), with its effective magnetic moment increasing to 3.75 μ_B_ (compared to 2.16 μ_B_ in Fe/N–C) (Fig. 6c, d). DFT calculations confirm the overlap between Fe *3d* and Mn *3d* orbitals, which optimizes the O_2_ adsorption bond length to 1.838 Å and achieves a moderate adsorption energy. This reduces the ORR energy barrier, resulting in a *E*_1/2_ of 0.928 V in an alkaline environment, far exceeding that of Fe SASCs. Moreover, after 8,000 potential cycles, *E*_1/2_ only lost 18 mV. These findings demonstrate that the synergistic spin configuration enhances spin matching efficiency and catalytic performance. Moreover, ADASCs enable directional transfer of spin-polarized charge, directly modulating the spin density distribution at active sites. Driven by electronegativity differences, electrons flow from the donor metal to the acceptor metal. This charge transfer is inherently spin-polarized, meaning electrons of a specific spin orientation are preferentially transferred. The result is not only a shift in *E*_d_ but also a change in the number of unpaired electrons at the metal center, facilitating a spin state transition. For instance, Li et al. predicted via theoretical calculations that the Fe–Ni dual-atom active center modulates the spin polarization of conducting electrons [104]. Specifically, constructing the Fe–Ni bimetallic active center introduces an electronegativity difference between Fe and Ni, which drives the directional electron transfer from Fe (electron donor) to Ni (electron acceptor). DFT calculations show that the spin magnetic moment of Fe decreases from 1.88 *μ*_B_ in its elemental state to 1.48 *μ*_B_ in with direct O–O cleavage, and the number of unpaired electrons is reduced, realizing optimization of the Fe spin state. Electrochemical test results show that the Fe–Ni atomic pair acts as a superior bifunctional catalyst for high-performance ORR and OER, with a very small potential difference of 0.691 V. Long-term stability test of ORR was performed by using the chronoamperometric measurement, after 20,000 s, the current is able to remain over 97%, much better than that of Pt/C (Fig. 6e). In addition, the asymmetric coordination environment in ADASCs disrupts the local symmetry of typical M–N_4_ structures, causing crystal field distortion. This distortion modifies the energy splitting between the *t*_*2g*_ and *e*_*g*_ orbitals. Concurrently, direct metal–metal bonding introduces additional orbital interactions that further influence electron distribution. The synergy between these effects (crystal field distortion and metal–metal interactions) collectively determines the final spin state of the active sites. For instance, Luo et al. enhanced OER activity and stability by introducing the 3*d* transition metal Mn into the symmetric CoOOH lattice [105]. This substitution lowers local symmetry and induces significant crystal field distortion, yielding a CoMnOOH phase with an optimized lattice structure. DFT calculations reveal that this lattice distortion minimizes the energy separation between the *d*_*xy*_ and *d*_*z*_^*2*^ orbitals of Co and alters the crystal field splitting energy. These electronic structure modifications facilitate electron population of the *e*_*g*_ orbitals, thereby tuning the spin state of Co sites. The optimized electronic structure reduces the energy barrier of the rate-determining step (RDS), ultimately improving overall OER kinetics (Fig. 6f, g).

Through three interconnected mechanisms: spin exchange reconstruction, spin-polarized charge transfer, and synergistic tuning of *d* orbital splitting, ADASCs achieve precise and cooperative optimization of the spin state at active sites. This capability not only enhances spin matching efficiency with reactants but also optimizes the reaction pathway from both thermodynamic (e.g., reducing reaction energy barrier) and kinetic (e.g., accelerating RDS) perspectives, thereby laying a foundation for boosting overall catalytic performance—representing a key advantage of ADASCs over conventional catalysts (e.g., SASCs, SDASCs).

It is noteworthy that spin configuration optimization strategies still exhibit certain limitations in regulating the catalytic performance of ADASC as follows: (1) Precise differentiation between bulk and surface phases, as well as active and inactive phases, remains challenging. Catalytic reactions occur on the catalyst surface, which inherently differs from the bulk phase. Furthermore, the spin behavior of different metal cations varies under distinct conditions and positions. During reactions, the surface phase is prone to reconstruction accompanied by dynamic spin state changes, which have yet to be fully explored. (2) The quantitative structure–activity relationship between spin states and catalytic performance remains ambiguous: Current regulation of spin configurations in ADASCs largely relies on empirical exploration, hindering the establishment of precise quantitative correlations between spin states (e.g., spin polarization degree, spin coupling modes) and catalytic activity, selectivity, or stability. (3) The impact of complex reaction conditions on spin-related electrocatalysis. Practical catalytic scenarios (e.g., fuel cell electrode reactions) often involve high temperatures, pressures, strong acidic/alkaline environments, and interference from impurity molecules. These typically constitute complex reaction systems prone to inducing spin rearrangement at ADASCs' active sites or metal atom agglomeration. This diminishes spin matching efficiency, thereby limiting their practical applications. Potential approaches to overcome these limitations include: (1) Integrating in situ X-ray magnetic circular dichroism (XMCD) with high-pressure electrochemical cells to precisely capture spin state differences between active, surface, and bulk phases, while simultaneously monitoring spin changes induced by surface phase reconstruction during reactions. (2) Enhancing in situ characterization and mechanism investigation systems by integrating techniques such as in situ synchrotron radiation and time-resolved spectroscopy. Integration of these techniques with machine learning algorithms to decipher the dynamic evolution of spin states can provide data-driven insights for strategy optimization. (3) Synergistic optimization of support microenvironment modification and active site protection. This includes introducing highly stable heteroatoms (P, S) to enhance metal–support interactions, suppressing spin reconstruction and atom agglomeration, or employing alloying modifications to improve catalyst magnetic stability. These approaches effectively overcome application bottlenecks in spin configuration optimization strategies, advancing the large-scale deployment of ADASCs in practical scenarios.

### Promoting Orbital Hybridization and Matching Energy Levels

During the electrocatalytic reaction process, orbital hybridization and energy level matching are core mechanisms and regulatory strategies that influence catalytic kinetics and selectivity [58, 106–109]. The essence of orbital hybridization lies in the effective overlap between the *d* orbitals of metal active sites and the molecular orbitals of reactants and intermediates; the degree of this overlap directly determines the efficiency of electron transfer—stronger overlap typically enhances electron transfer rate. Energy level matching is achieved by regulating the relative positions between the metal *E*_d_ and the adsorption energy levels of intermediates, thereby optimizing the adsorption energy of intermediates [77, 110]. According to the Sabatier principle, excessively strong adsorption energy will hinder product desorption and reduce catalytic efficiency; conversely, excessively weak adsorption will make it difficult to activate reactants, limiting the further progression of the reaction. Therefore, precise energy level matching is also crucial for synergistically balancing catalytic activity and selectivity in target reactions.

**Fig. 7:**
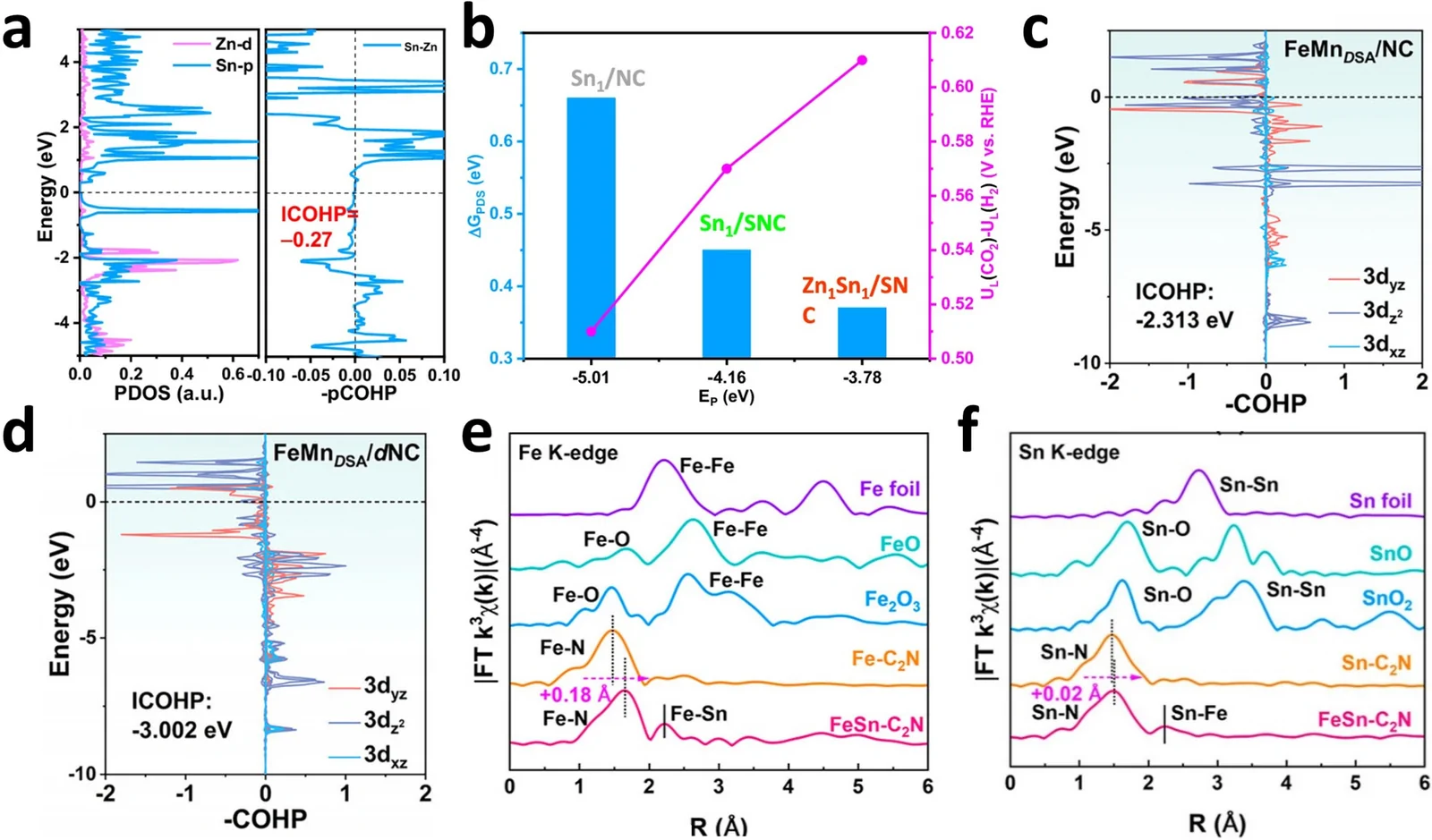
ADASCs optimize the hybridization and energy level matching. **a** PDOS and COHP data of Zn_1_Sn_1_/SNC DACs. **b** Comparison of Δ*G*_PDS_ and U_L_(CO_2_)-U_L_(H_2_). Reproduced with permission [113]. Copyright 2025, Springer Nature. **c, d** pCOHP of *OOH adsorbed for FeMnDSA/NC and FeMnDSA/dNC. Reproduced with permission [77]. Copyright 2025, Wiley-VCH. **e, f** EXAFS spectra of FeSn–C_2_N. Reproduced with permission [114]. Copyright 2024, American Chemical Society

SASCs and SDASCs are limited by their single-site configurations, resulting in fixed metal orbital interaction modes that cannot meet the adsorption/desorption requirements of diverse intermediates in multi-step reactions. The asymmetric active centers of ADASCs benefit from inherent differences in *d*-electron configurations, electronegativities, and atomic radii. Through interatomic orbital coupling, they provide a structural basis for the precise regulation of orbital hybridization strength and *E*_d_ positions. Their asymmetric coordination environment can tailor the electron cloud distribution of metal *d* orbitals to enhance hybridization [111, 112]. For example, Peng et al. successfully synthesized asymmetric Zn–Sn dual-atom sites via a ligand co-etching strategy, where Zn and Sn centers are coordinated with N and S heteroatoms, respectively [113]. EXAFS fitting results confirmed the formation of Zn–Sn bonds with a bond length of 2.22 Å, verifying the atomic proximity of the dual-atom pair. PDOS analysis further revealed significant overlap between the Zn 3*d* and Sn 5*p* orbitals, directly confirming interatomic orbital coupling. The asymmetric coordination environment of the catalyst altered the metal orbital electron cloud distribution and enhanced *p–d* hybridization. DFT calculations indicated that partial S doping into disrupted local symmetry, leading to reduced electron density at the Sn center (Fig. 7a). Consequently, the asymmetric Zn–Sn sites enhanced the adsorption strength of the ^*^HCOO^−^ intermediate in CO_2_RR, enabling a formate Faraday efficiency of 94.6% in the H-type cell (Fig. 7b). In addition, heteronuclear ADASCs exhibit lattice mismatch derived from atomic radius differences, which is key to modulating *d* orbital overlap degree and thus tuning the *d*-band width (a key descriptor of energy level distribution). For example, Chen et al. constructed a defect-engineered FeMn dual-atom catalyst on N-doped porous carbon via a trinuclear defect trapping strategy [77]. This strategy stabilized FeMn pairs while optimizing the electronic structure to the Sabatier optimum, significantly enhancing ORR performance. Theoretical calculations demonstrated that defect-mediated coordination triggered charge redistribution around Fe–Mn dual-atom centers, suppressing antibonding orbital filling and enhancing Fe 3*d*_*z*_^*2*^–O *2p* hybridization (Fig. 7c, d). This modulation weakened the O–O bonding via optimized ^*^OOH adsorption, thereby accelerating ORR kinetics. Notably, heteronuclear metals typically exhibit distinct electronegativity differences, which drive directional electron transfer from the less electronegative metal to the more electronegative one—this charge redistribution is conducive to regulating the *E*_d_ and optimizing energy level matching. Furthermore, asymmetric configurations induce crystal field distortion, altering *d* orbital splitting energy to further tune orbital energy levels and synergistically enhance hybridization/energy level matching effects. For example, Wang et al. prepared a FeSn-C_2_N by embedding asymmetric Fe–Sn dual-atom sites in two-dimensional C_2_N nanosheet [114]. EXAFS confirmed Fe–Sn bonds (2.54–2.57 Å), and PDOS analysis revealed Fe *3d*-Sn *5p* coupling. The asymmetric coordination environment tailored Fe *d* orbital electron clouds, enhanced *p–d* hybridization, and optimized the ^*^OH adsorption energy. Under alkaline conditions, the *E*_1/2_ of the ORR reached 0.914 V, surpassing Fe-C_2_N and Sn-C_2_N In the ZAB, it operates stably for 320 h at a current density of 10 mA cm^−2^ (Fig. 7e, f).

In summary, through orbital hybridization and energy level matching, ADASCs can precisely regulate the electronic state of catalytic centers to optimize the adsorption energy of key intermediates, and strengthen dual-site synergistic effects to enhance reaction kinetics. However, they suffer from limitations including difficulty in precise regulation, easy structural instability during reactions, and lagging theoretical understanding and characterization techniques. In the future, it is essential to realize directional modulation of orbital hybridization and energy levels through advanced precise synthesis strategies. Concurrently, integrating in situ multi-characterization technique coupling and machine learning-assisted advances in theoretical modeling will bridge the gap between structure, mechanism, and performance, thereby accelerating their performance optimization for target reactions and practical application in electrochemical devices.

### Regulating Charge States and Electrical Conductivity

In electrocatalytic materials, the charge state refers to the characteristics of electron distribution at the active sites within the material, such as the overall charge property, the uniformity of local charge density, and the degree of polarization. Its essence is a deviation of the electron cloud density at active sites from the neutral state, driven by factors such as the coordination environment, electronegativity differences between metals in heteronuclear diatomic pairs, and metal–support interactions [115], and this deviation directly modulates the electron donation/acceptance ability of active sites. Conductivity, on the other hand, describes the electron transfer rate within the catalyst bulk and across the catalyst–electrolyte interface. Low conductivity elevates electron transfer resistance at these interfaces, resulting in increased reaction overpotential and retarded catalytic kinetics [116]. Therefore, the charge state modulates the charge density of active sites by tuning the adsorption energy of key reaction intermediates, thereby altering the reaction energy barrier. In contrast, conductivity primarily governs the electron transfer efficiency during the electrocatalytic process [117, 118]. These two properties synergistically facilitate the entire electron transfer pathway in electrocatalysis and act as a core link for tuning the electronic structure of active sites and optimizing catalytic reaction kinetics.

**Fig. 8:**
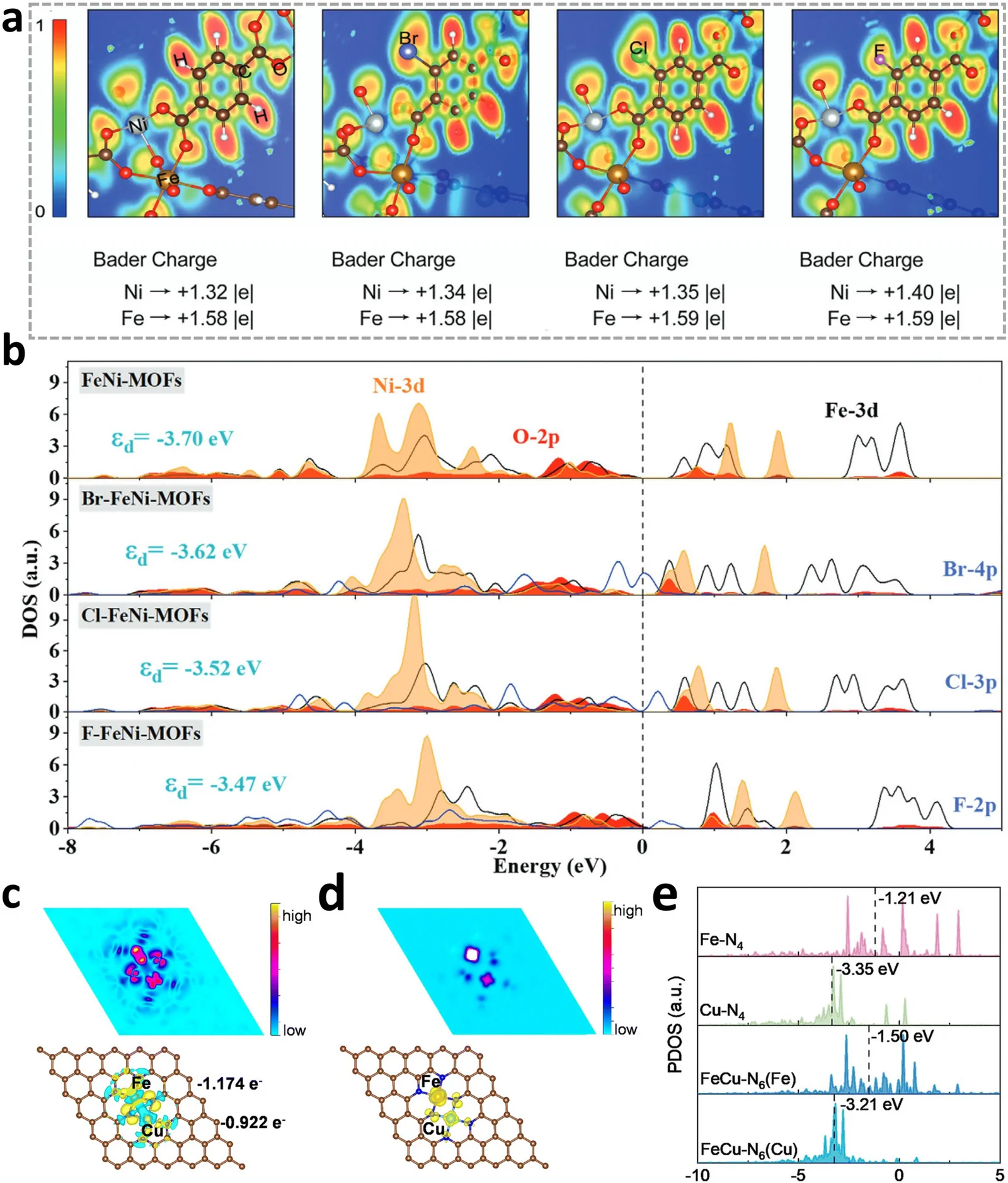
ADASCs optimize the charge state and electrical conductivity. **a** ELF and b PDOS of FeNiMOF, and X-FeNi-MOFs (X = Br, Cl, and F). Reproduced with permission [120]. Copyright 2024, Wiley-VCH. **c** Bader charge analysis and **d** spin density distribution and channel in FeCu-N6. **e** PDOS and *E*_d_ positions for metal sites in different models. Reproduced with permission [78]. Copyright 2025, The Royal Society of Chemistry

Compared with SASCs (with fixed charge distribution at single M–N_4_ sites) and SDASCs (with limited intersite orbital interaction at the homonuclear metal centers and constrained dimensionality for conductivity modulation), the asymmetric metal centers of ADASCs possess inherent electronic differences. Moreover, the asymmetric coordination environment further breaks the symmetry of charge distribution, providing multiple degrees of freedom for charge state regulation. Concurrently, the interatomic orbital coupling between heterometals and strong metal–support interactions (SMSI) construct a continuous electron transfer channel, significantly reducing electron transfer resistance and improving overall conductivity [119]. Furthermore, the inherent electronegativity differences between distinct metal centers in most ADASCs drive directional electron transfer, forming polar bonds and altering the local charge density distribution at active sites. For instance, Hu et al. constructed an asymmetric coordination environment by modifying FeNi-MOFs with halogens (F, Cl, Br), which provides multiple degrees of freedom for charge state regulation [120]. DFT calculations show that as the electronegativity of halogens increases from Br (2.96) and Cl (3.16) to F (3.98), the Bader charge of Ni increases from 1.32 e^−^ to 1.40 e^−^ (Fig. 8a), this charge variation confirms the directional electron transfer driven by electronegativity differences. PDOS analysis indicates that the band centers of Ni 3*d* orbitals shift closer to the Fermi level, enhancing the coupling of metal orbitals (Fig. 8b). Electrochemical tests confirm that F-FeNi-MOFs exhibit a double-layer capacitance of 1.82 mF cm^−2^, along with the lowest charge transfer resistance and optimized conductivity. Consequently, its OER overpotential is only 218 mV at a current density of 10 mA cm^−2^. And there is negligible decay after the operation of 150 h. Ao et al. synthesized the FeCu-NC ADASCs via a host–guest encapsulation strategy [78]. Bader charge analysis revealed that electrons are directionally transferred from Fe to Cu, forming polar bonds and altering the local charge density of Fe active sites (Fig. 8c, d). EXAFS characterization confirmed an Fe–Cu bond length of 2.4 Å. PDOS analysis showed that the *d* orbital coupling between Fe and Cu shifts the Fe *E*_d_ downward (Fig. 8e), which optimizes the adsorption energy of ORR intermediates. Additionally, the strong interaction between the metals and the N-doped carbon support constructs a continuous electron transfer channel, reducing electron transfer resistance. As a result, FeCu-NC ADASCs exhibit a *E*_1/2_ of 0.918 V for the ORR in alkaline media, which is significantly higher than those of Fe-NC and Cu-NC SASCs. Moreover, the catalyst demonstrates remarkable stability with negligible degradation after accelerated degradation testing.

In conclusion, the optimization of electrocatalytic performance by ADASCs requires multi-dimensional synergistic regulation. Structurally, physical confinement is achieved through the formation of metal–metal covalent bonds between heteronuclear metals and bridging via shared ligands; combined with electronic coupling effects, this suppresses metal atom agglomeration and enables stable atomic dispersion even at high metal loading. At the electronic level, ADASCs with heteronuclear metal centers optimize the adsorption energy of key intermediates via strain effects and spin coupling. In contrast, homonuclear ADASCs achieve analogous electronic regulation by tuning the *E*_d_ and enhancing orbital hybridization via asymmetric ligands. In terms of charge transfer, the electronegativity difference between asymmetric metal centers drives directional electron transfer, forming polar bonds at the metal active site interface. This reduces electron transfer resistance and improves catalytic kinetics. This multi-dimensional synergy endows ADASCs with a more favorable catalytic structure than SASCs and SDASCs, thereby significantly boosting catalytic performance in target reactions. The aforementioned multi-dimensional synergistic regulation mechanism provides a core guiding logic for the precise construction of ADASCs. Its essence lies in realizing the synergistic optimization of structural, electronic, and charge transfer properties through the directional regulation of the asymmetry of coordination environments and metal centers. This logic clarifies that the construction of ADASCs must focus on two core directions—coordination environment modification and metal center design—to fully unleash their structural advantages and translate them into excellent catalytic performance.

## Strategies for Constructing Asymmetric Structures

The asymmetric structural advantages of ADASCs lay a crucial foundation for enhancing catalytic performance in electrochemical reactions. To fully exploit these structural advantages for practical electrocatalytic applications, it is essential to clarify the specific construction strategies of asymmetric structures in ADASCs. According to recent studies, the asymmetric structures of ADASCs primarily originate from the dual regulation of coordination environments and metal centers [121, 122]. Among these regulatory approaches, the asymmetry of the coordination environment is mainly achieved via nonmetallic heteroatom modification: Introducing heteroatoms (e.g., S, P, O) to replace partial N atoms in the M–N_4_ framework breaks the symmetric coordination structure, generating asymmetric configurations. This modification further induces local charge rearrangement and optimizes the coordination environment of active sites [123]. The asymmetry of metal active centers relies on the rational design of heteronuclear metal atoms: Distinct metals with inherent differences in electronegativity, atomic radius, and *d*-electron configuration are rationally designed to construct active sites with dual asymmetry in both electronic and geometric configurations [124–126]. Thus, this section focuses on asymmetric active site construction strategies in ADASCs, which are categorized into two key approaches: nonmetallic heteroatom modification and heteronuclear metal design.

### Nonmetallic Heteroatom Modification

**Fig. 9:**
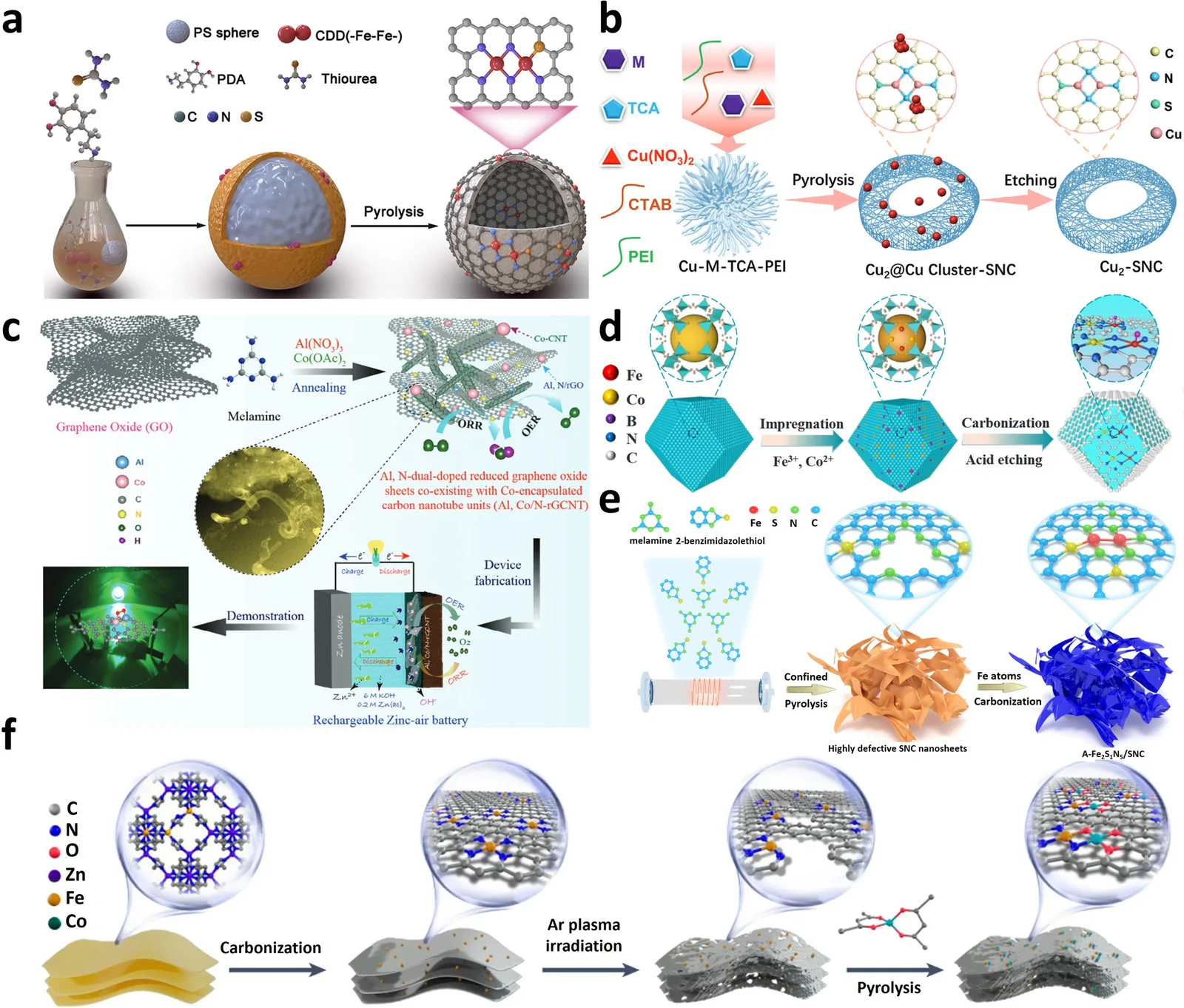
Constructing asymmetric structures via nonmetallic heteroatom. **a** Schematic synthesis of Fe_2_S_1_N_5_/SNC. Reproduced with permission [129]. Copyright 2024, The Royal Society of Chemistry. **b** Schematic synthesis of Cu_2_-SNC. Reproduced with permission [131]. Copyright 2024, Wiley-VCH. **c** Schematic synthesis of the Al,Co/N-rGCNT electrocatalyst. Reproduced with permission [130]. Copyright 2024, Wiley-VCH. **d** Schematic synthesis of Fe/Co-B-NC. Reproduced with permission [132]. Copyright 2025, Elsevier. **e** Schematic synthesis of A-Fe_2_S_1_N_5_/SNC. Reproduced with permission [46]. Copyright 2024, Springer Nature. **f** Schematic synthesis of FeCo-N_3_O_3_@C. Reproduced with permission [56]. Copyright 2024, Springer Nature

Among DASCs, the construction of asymmetric coordination environments for ADASCs typically relies on the introduction of nonmetallic heteroatoms (e.g., S, P, O, B) with distinct atomic sizes and electronegativities [127, 128]. These heteroatoms act through two pathways: either replacing partial coordinating atoms in traditional symmetric motifs (e.g., M–N_4_) or embedding into the carbon support framework. Both approaches construct asymmetric environments at the coordination shell level, inducing charge redistribution and orbital reconstruction of metal active sites. This strategy not only enhances the anchoring of isolated metal dimers to suppress agglomeration but also optimizes intermediate adsorption and accelerates catalytic reactions kinetics. Currently, the precursor preselection method enables relatively precise control over nonmetallic heteroatom doping for ADASCs synthesis. During precursor preparation, nonmetallic heteroatom precursors are mixed with metal precursors and supports; pyrolysis then drives coordination between heteroatoms and metal centers, forming asymmetric coordination configurations. For example, Li et al. utilized bis(dicarbonylcyclopentadienyliron) (CDD, a dinuclear Fe precursor) for catalyst synthesis [129]. During dopamine polymerization, CDD thiourea (S source) were in situ encapsulated within the pores of polydopamine (PDA), forming S/CDD/PS@PDA core–shell structure. Pyrolysis converted PDA into a N-doped porous carbon shell (N–C–H) (serves as the active site support), successfully preparing a catalyst with charge-asymmetric diatomic sites (Fe_2_S_1_N_5_/SNC) (Fig. 9a). HAADF-STEM observations revealed that metal atoms in Fe_2_S_1_N_5_/SNC mainly exist as paired diatomic species. EXAFS spectra confirmed the structure of its charge-asymmetric diatomic active sites. Further analysis demonstrated that the two charge-asymmetric Fe sites, designed via the precursor preselection method, effectively optimize the adsorption/desorption behavior of the ^*^OH intermediate during the ORR. Kharabe et al. conducted high-temperature pyrolysis and secondary annealing on a premixed precursor system (graphene oxide (GO), Co(OAc)_2_, Al(NO_3_)_3_, melamine), successfully preparing a bifunctional electrocatalyst (Al,Co/N-rGCNT) [130]. This catalyst features the coexistence of Al and N dual-doped reduced graphene (Al,N-rGO) and Co-encapsulated carbon nanotubes (Co-CNT), with an asymmetric Al-N-Co-N_x_ coordination configuration formed (Fig. 9c). For ADASCs synthesized via this method, heteroatoms and metal centers coordinate synchronously during pyrolysis, forming strong interactions that prevent metal agglomeration caused by heteroatom detachment in subsequent processes. However, once pyrolysis is completed, the coordination structure in the catalyst becomes fixed, precluding gradient regulation of heteroatom proportions. Compared with the precursor preselection method, the post-modification etching method enables gradient optimization of heteroatom doping proportions. Under appropriate conditions, it can modify specific metal sites by adjusting etching time or heteroatom precursor concentration. For example, Chen et al. synthesized the Cu_2_-SNC catalyst via a reverse micelle assembly–pyrolysis strategy [131]. First, a micellar polymer was prepared, this polymer was then transferred to a tube furnace for pyrolysis, yielding a catalyst precursor containing isolated Cu atoms, Cu nanoparticles, and Cu oxides. Etching with HNO_3_ afforded Cu_2_-SNC with a nest-like structure dominated by dual Cu atomic sites. This asymmetric structure consists of porous walls and a hollow architecture, where the asymmetric coordination of dual Cu sites relies on S substitution. During synthesis, the dosage of polyethyleneimine (PEI) is critical: A high PEI concentration leads to hollow structure closure, while a low PEI concentration hinders hollow structure formation, resulting in bulk materials (Fig. 9b). DFT calculations revealed that S doping induces changes in the electronic state distribution of Cu, forming an asymmetric electron cloud. This promoting the asymmetric C–C coupling pathway, preferentially generating ethanol while suppressing ethylene formation. Dang et al. etched pre-synthesized PA@Fe/Co-ZIF-8, successfully designing a boron-regulated Fe–Co dual-atom catalyst (Fe/Co–B–NC) (Fig. 9d) [132]. HAADF-STEM characterizations demonstrated that dense Fe-Co bimetallic atomic pairs are distributed on the carbon layer of Fe/Co-B-NC catalyst. XAS fitting analysis revealed that each Fe atom coordinates with four N atoms and one B atom, while adjacent Fe and Co atoms are linked by two shared N atoms. Further analysis confirmed that this diatomic site configuration effectively weakens the adsorption strength of the oxygen-containing intermediate (^*^OH), significantly reducing the reaction energy barrier and thereby enhancing the activity and stability of Fe/Co–B–NC. Nevertheless, since heteroatoms combine with metal atoms in the later synthesis stage, the coordination strength is weaker than that of in situ doping, potentially leading to metal detachment during reactions. To address this issue, metal–support interactions can be further enhanced: Reducing the surface free energy of metal atoms and increasing the kinetic barrier for atomic diffusion/agglomeration enable regulation of active site dispersion. The support heteroatom doping method allows simultaneous embedding of heteroatoms into the carbon framework. This not only enables heteroatoms to participate in metal coordination and strengthen metal–support interactions but also optimizes support electrical conductivity to enhance catalytic efficiency. For example, Zhang et al. first ground 2-benzimidazolethiol and melamine at a mass ratio into a homogeneous mixture, then conducted pyrolysis in a flowing inert atmosphere [46]. Active C atoms in melamine are likely to form C–S–C covalent bonds with thiol groups; these bonds further transform into a carbon nitride (NC) structure with spontaneous defect formation. During the thermal polymerization of 2-benzimidazolethiol, NC was encapsulated via C–S–C bond connection; pyrolysis converted the constructed carbon skeleton into S, N-co-doped carbon nanosheets. Subsequently, Fe atoms were anchored via S and N coordination to form asymmetric diatomic sites, successfully preparing an asymmetrically coordinated diatomic iron catalyst (A-Fe_2_S_1_N_5_/SNC) (Fig. 9e). DFT calculations revealed that the enhanced OER activity of the A-Fe_2_S_1_N_5_/SNC originates from the spontaneous electric polarization of Fe diatomic sites. This catalyst exhibited excellent water oxidation performance in alkaline medium: Its overpotential at 10 mA cm^−2^ was only 193 mV, which is lower than that of commercial RuO_2_. However, this method suffers from a critical limitation: The coordination between nonmetallic heteroatoms and metal centers is highly stochastic, making it difficult to precisely control the heteroatom coordination state of each active site. Additionally, due to challenges in precisely regulating the coordination environment of two distinct metal atoms, most heteronuclear ADASCs developed in recent years only contain one type of coordinating anion and maintain a relatively symmetric coordination structure. This uniformity limits the further improvement of catalytic performance.

To address the aforementioned issues and fully leverage ADASCs in complex multi-step catalytic systems, recent studies have revealed that diverse nonmetallic heteroatoms can act as anionic ligands to coordinate two distinct metals, constructing Janus bimetallic sites with asymmetric coordination configurations—a structural feature that breaks the symmetry of active centers to enhance catalytic versatility, which is a key advantage for boosting catalytic performance [133]. Tang et al. employed 2D MOF nanosheets as precursors to pre-prepare Fe–N_4_ single atoms catalysts (FeN_4_@C) [56]. Subsequently, Ar plasma irradiation was applied to create atomic vacancies; notably, the high surface energy derived from N/O co-doping promoted preferential vacancy formation around Fe atoms, thus converting FeN_4_@C into defect-rich FeN_3_@C (d-FeN_3_@C) (Fig. 9f). To construct the Janus structure, the Co–O_3_ moiety was precisely anchored into the Fe-adjacent vacancies of d-FeN_3_@C via low-temperature pyrolysis of Co(acac)_2_. This process successfully yielded the Janus FeCo–N_3_O_3_@C catalyst, where Fe and Co atoms are coordinated with N and O atoms respectively and linked via bridging N/O atoms, realizing the asymmetric coordination that defines the Janus sites. In situ synchrotron radiation Fourier transform infrared (SR-FTIR) spectroscopy, XANES combined with DFT calculations confirmed that the Fe–N_3_ and Co–O_3_ moieties serve as dedicated active sites for the ORR and OER, respectively, and facilitate the 4e^−^ pathway. The enhanced bifunctional activity stems from N/O heteroatoms regulating the 3*d* orbital filling of Fe/Co: N with high electronegativity downshifts the Fe *E*_d_ to weaken ^*^O adsorption (optimizing the ORR RDS of ^*^O desorption), while O coordinates to upshift the Co *E*_d_ to strengthen ^*^OOH formation (promoting the OER RDS). This strategy provides a feasible route for designing advanced carbon-based diatomic catalysts and offers theoretical guidance for multi-functional catalyst development.

In conclusion, constructing asymmetric diatomic site catalysts via regulated nonmetallic heteroatom introduction features core advantages: precise tailoring of the electronic/geometric structures of active sites, expanded catalytic multi-functionality, enhanced structural stability, and improved electron transfer efficiency, thereby solving key challenges in multi-step complex catalysis. However, this strategy still faces limitations, including imprecise control over the doping content and spatial distribution of nonmetallic heteroatoms, structural collapse under harsh reaction conditions, high costs of certain heteroatom precursors coupled with difficulties in large-scale synthesis, and ambiguous synergistic mechanisms between nonmetallic heteroatoms and bimetallic centers. To mitigate these issues, feasible strategies are proposed as follows: optimizing precursor design at the molecular level and refining synthesis processes to regulate heteroatom distribution; reinforcing structural stability via secondary annealing or protective layer coating; developing low-cost nonmetallic heteroatom sources and simplifying preparation workflows; and deepening mechanistic research by combining in situ characterization techniques with theoretical calculations. These measures are expected to accelerate the practical application of such Janus-type diatomic site catalysts in energy conversion systems.

### Heteronuclear Metal Design

Since the synthesis of DASCs is more challenging than that of SASCs, effective methods for regulating the formation of dual-atom sites are urgently required. Heteronuclear DASCs offer more elemental combinations compared to homonuclear DASCs, thereby opening up greater possibilities for the application of DASCs in catalytic reactions. Among DASCs, doping distinct metal atoms can break the inherent symmetry of active centers, leading to the formation of asymmetric dual-atom configurations and thus enabling the synthesis of ADASCs. This heteronuclear metal modification strategy alters the symmetric electronic structure of SASCs through the interaction between the primary active center metal (M_1_) and the doped metal atom (M_2_), which not only enhances catalytic activity but also improves reaction kinetics. Essentially, it leverages metals with inherent differences in electronegativity, atomic radius, and *d*-electron configuration to construct active sites with dual asymmetry in both electronic and geometric configurations [134–136].

**Fig. 10:**
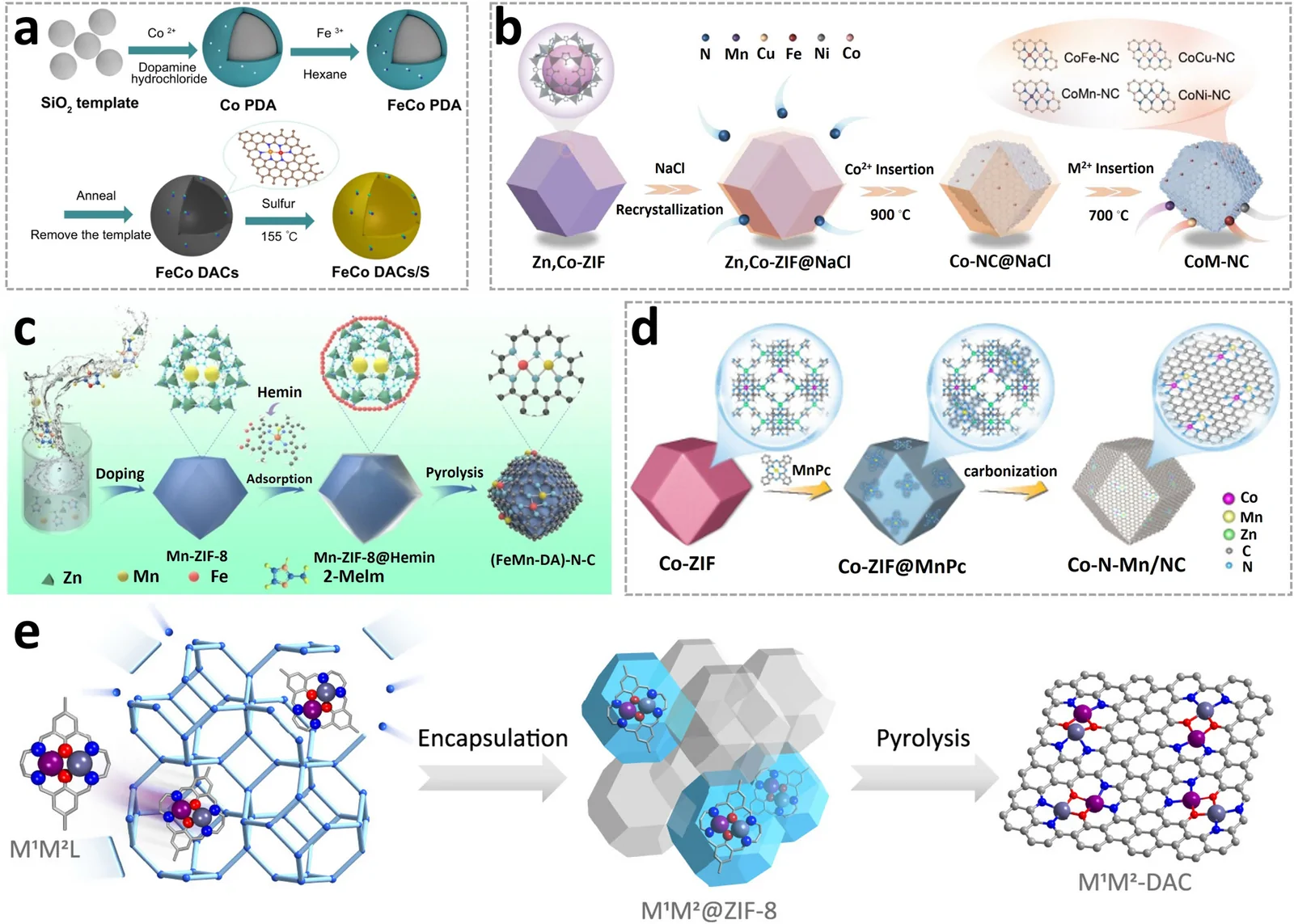
Constructing asymmetric structures via heteronuclear metal atoms. **a** Schematic synthesis of FeCo DACs/S. Reproduced with permission [140]. Copyright 2023, Springer Nature. **b** Schematic synthesis of CoM-NC. Reproduced with permission [141]. Copyright 2025, Wiley-VCH. **c** Schematic synthesis of (FeMn-DA)-N-C. Reproduced with permission [142]. Copyright 2025, Springer Nature. **d** Schematic synthesis of Co–N–Mn/NC catalyst. Reproduced with permission [143]. Copyright 2025, Springer Nature. **e** Schematic synthesis of FeCu-DAC. Reproduced with permission [144]. Copyright 2023, American Chemical Society

Extensive studies have been conducted on the synthesis of ADASCs with heteronuclear metal centers [137–139]. The two-step method is a commonly used strategy for constructing the asymmetric heteronuclear metal structure of ADASCs: By precisely depositing a second metal onto a pre-prepared single-metal-site support, the molar ratio and interatomic spacing of the two metals can be effectively controlled to avoid homonuclear agglomeration. For example, Sun et al. used SiO_2_ as a hard template, under alkaline conditions, dopamine hydrochloride was self-polymerized to form a polydopamine (PDA) coating [140], while cobalt nitrate was added to adsorb Co^2+^ on the PDA surface via coordination, yielding a single Co-metal precursor (Co-PDA). Based on this Co-PDA precursor, an iron nitrate solution was added dropwise; utilizing the chemical adsorption of Co sites, polar Fe species were directionally bound to the Co-PDA surface, forming an Fe-Co-PDA bimetallic precursor. Subsequently, through annealing in an NH_3_ atmosphere, removal of the SiO_2_ template, and acid treatment, Fe-Co DACs were finally obtained, realizing the construction of heteronuclear metal centers (Fig. 10a). Electrochemical tests, combined with in situ Raman spectroscopy and theoretical calculations, indicated that this unique dual-site structure not only enhances the anchoring ability for lithium polysulfides (LiPSs) but also accelerates the reaction kinetics of LiPSs conversion and Li_2_S decomposition, achieving efficient bifunctional catalytic effects during both charging and discharging processes. Wang et al. first obtained a Co single-metal support (CoNC) through ZIF pyrolysis and template removal [141], then added metal salts (M = Fe, Cu, Mn, Ni) for secondary pyrolysis to form diatomic sites, successfully synthesizing a series of CoM-NC catalysts (Fig. 10b). A series of characterizations has confirmed that this stepwise anchoring strategy effectively avoids the agglomeration issue arising from the simultaneous introduction of bimetals, enabling the uniform dispersion of Co-M atomic pairs. Performance tests and DFT calculations demonstrate that the intermediate electronegativity of Fe promotes stronger electronic coupling with adjacent Co atoms, optimizing the adsorption of peroxymonosulfate (PMS) at the active sites and thus endowing CoFe-NC with excellent degradation activity for organic pollutants. However, this method is prone to uneven local distribution of metal ions, which may lead to atomic agglomeration during the deposition process. Additionally, when ZIF nanocrystals are used as precursors, most of the active sites in the resulting M–N-C catalysts after high-temperature carbonization are buried within the carbon matrix, reducing site accessibility. To address this issue, the surface adsorption precursor preparation strategy can optimize the exposure degree of active sites and improve site accessibility. For example, Zhang et al. used Mn-ZIF-8 as a carrier to adsorb hemin (15.1 × 15.3 × 10.6 Å^3^; ZIF cavity diameter ≈ 11.6 Å) [142], which is larger than the ZIF cavity, on the outer surface of the crystal. After pyrolysis, an atomically dispersed Fe–Mn dual-atom site ORR catalyst ((FeMn-DA)-N-C) was obtained, effectively enhancing site accessibility (Fig. 10c). DFT calculations indicated that the introduction of Mn atoms modulated the electronic structure of the active Fe sites, optimizing the adsorption energy of reaction intermediates and the *E*_d_ of the Fe center. As a more straightforward strategy for constructing ADASCs with heteronuclear metal atoms, the dual-solvent directed adsorption method utilizes the liquid–liquid interface effect to enable metal ions to be directionally adsorbed onto the support surface based on their hydrophilic–hydrophobic properties. This achieves the ordered arrangement of metal atoms and reduces random distribution. For instance, Liu et al. proposed and validated a geometric–electronic coupled design principle [143]: Using the interatomic distance between the two metals and the magnetic moment of the active center as the two main axes, a machine learning-derived "hot spot map" was plotted. This map exhibits a narrow high-activity region, directly guiding the rapid screening and experimental synthesis of DASCs. The predicted optimal catalyst (Co–N-Mn/NC) was synthesized experimentally. First, pre-synthesized Co-ZIF-8 was dispersed in n-hexane, where Co-ZIF-8 maintained stable dispersion due to its hydrophobicity. Then, manganese phthalocyanine (MnPc) was dissolved in ethanol, and the resulting solution was added dropwise to the n-hexane system under ultrasonication and stirring. Utilizing the hydrophilic–hydrophobic difference at the liquid–liquid interface, MnPc was directionally adsorbed on the surface of Co-ZIF-8 to form the MnPc@Co-ZIF-8 precursor, which was subsequently pyrolyzed at 950 °C in N_2_ (Fig. 10d). EXAFS spectroscopy and its fitting results revealed that the coupling of adjacent Co/Mn atoms modifies the local coordination environment. This catalyst exhibits a *E*_1/2_ of 0.90 V in alkaline electrolyte, outperforming commercial Pt/C, with superior durability and methanol poisoning resistance. Nevertheless, limited by immiscible solvent systems (e.g., n-hexane–water), the selection of supports is restricted, the metal loading is relatively low, and the solvent must be removed in subsequent steps to prevent residues from affecting catalytic performance. Owing to its self-limiting growth characteristic, which allows for relatively precise regulation of the bond length and coordination environment between heteronuclear metal atoms, atomic layer deposition (ALD) achieves improved atomic-level precision. For example, Zhang et al. first prepared a bimetal-containing Robson-type macrocyclic complex [144]. This complex has a pre-assembled bimetallic center in its molecular structure and a stable coordination configuration of [M_1_M_2_N_4_O_2_]. Subsequently, the macrocyclic complex was dispersed and encapsulated into ZIF-8 to form an M_1_M_2_L@ZIF-8 composite; as ZIF-8 possesses six-membered ring windows with a diameter of 0.34 nm, which can encapsulate the bimetallic macrocyclic complex within its pores, this spatial confinement effectively prevents the agglomeration of bimetallic precursors during pyrolysis. Pyrolysis was then carried out at 900 °C to synthesize ADASCs with heteronuclear metal centers (e.g., FeCu-DAC, FeNi-DAC) (Fig. 10e). Among these, FeCu-ADAC unlocked an unconventional reaction pathway during the oxygen reduction process: Through this pathway, the by-product H_2_O_2_ is mainly reduced to H_2_O rather than reactive oxygen species. Consequently, FeCu-ADAC exhibits unprecedented durability in acidic medium, with a Δ*E*_1/2_ of only 5 mV after 10,000 potential cycles. However, ALD requires specialized equipment and has low single-batch output, resulting in poor potential for large-scale applications.

Overall, the strategy of fabricating ADASCs via regulating heteronuclear metal atom introduction exhibits distinct advantages: It can precisely modulate the electronic structure, adsorption energy of reaction intermediates, and reaction kinetics of active sites through the electronic coupling and geometric synergy between heteronuclear metals. This not only significantly enhances catalytic selectivity and activity, but also enables the ordered dispersion of active sites by leveraging the unique structural features of supports (e.g., MOFs). However, this method also has notable limitations: (1) The structural evolution of ADASCs during the synthesis process remain unclear, and this lack of understanding leads to insufficient precise regulation, which tends to result in atomic agglomeration or uneven distribution of active sites; (2) Some preparation strategies are constrained by specialized equipment, involve complex multi-step processes, and are limited by low metal loading; (3) High-temperature treatment is prone to causing the collapse of the support structure, which impairs active site accessibility and long-term catalyst stability. To address these issues, potential solutions include: (1) Developing machine learning-based precise design methods combined with in situ characterization techniques to clarify the structural evolution mechanisms during synthesis; (2) Optimizing the support structure design and preparation processes, and adopting targeted strategies such as stepwise anchoring, spatial confinement, or dual-solvent directed adsorption to inhibit atomic agglomeration and ensure uniform active site distribution; (3) Advancing green preparation technologies to reduce dependence on specialized equipment and simplify procedures, while exploring synthetic routes under mild conditions to minimize high-temperature-induced damage to supports and active sites. These measures are expected to achieve dual breakthroughs in the precise regulation of ADAS structures and the large-scale practical application of such catalysts.

In conclusion, in constructing the asymmetric structure of ADASCs, the various strategies reported thus far (e.g., stepwise anchoring, dual-solvent directed adsorption, and atomic layer deposition) are not isolated; instead, they require integrated and synergistic optimization. Only through such collaborative regulation can the advantages of the asymmetric structure be maximized, thereby more effectively overcoming the performance bottlenecks of traditional catalysts. Nevertheless, current construction strategies still have notable limitations: for instance, the structural evolution details during ADASCs synthesis remain unclear, and the lack of precise control over these processes often leads to issues such as heteronuclear bimetallic active site agglomeration and uneven dispersion, rather than well-dispersed active sites. Thus, future research should continue to focus on advanced asymmetric structure construction strategies—such as machine learning-guided precursor design and mild-condition synthesis processes—to achieve dual breakthroughs: the precise regulation of ADAS structures and the scalable application of ADAS-based catalysts in practical scenarios.

## Advanced Characterization Techniques for Dual-Atom Site Catalysts

**Fig. 11:**
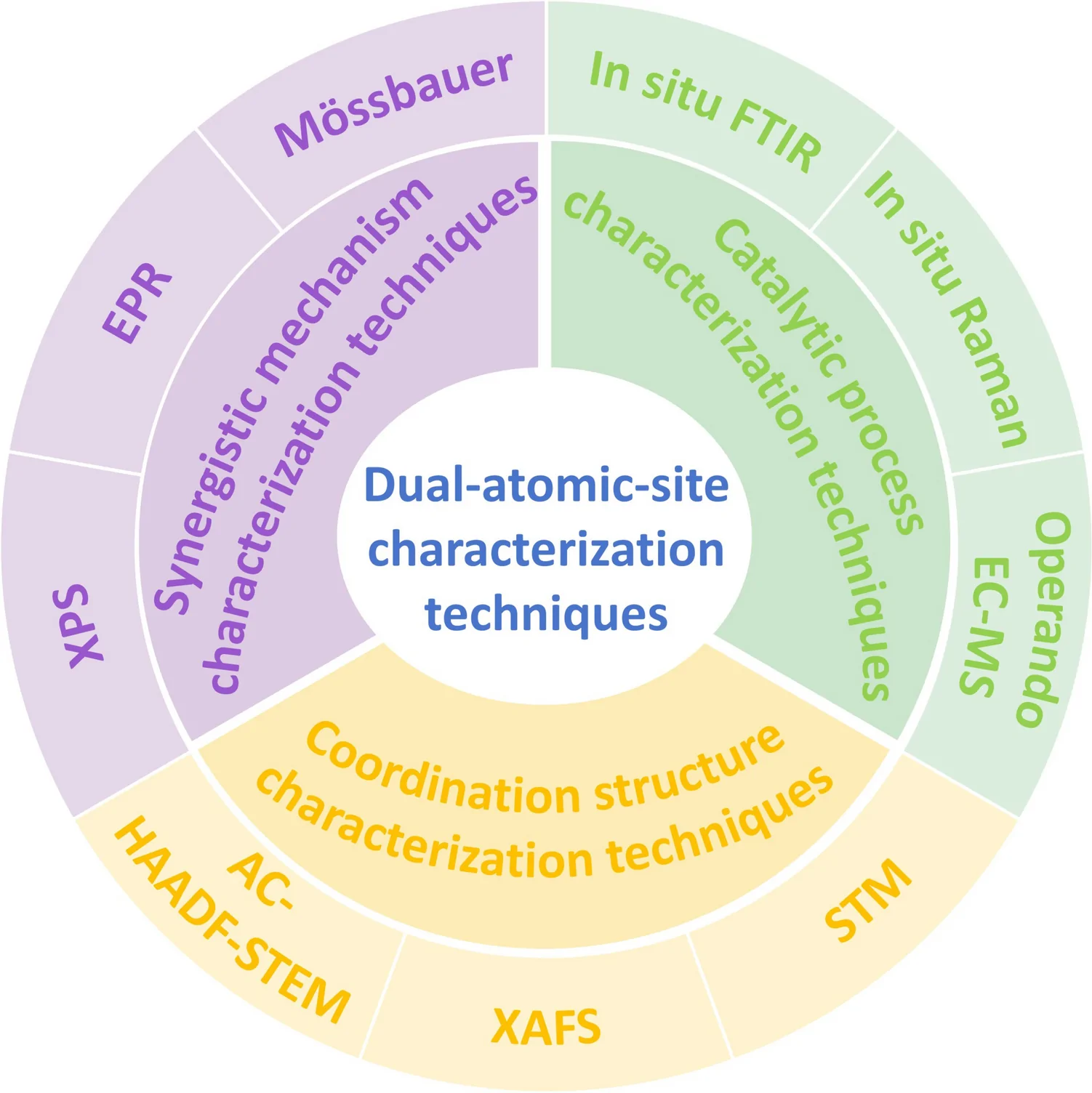
Advanced characterization techniques for dual-atomic-site catalysts

As a high-performance derivative of SASCs, DASCs derive their core advantages from the synergistic effects of metal atom pairs, precisely tailored coordination environments, and unique electronic structures [145, 146]. However, the intrinsic properties of DASCs differ fundamentally from those of traditional catalytic materials, rendering conventional characterization techniques inadequate for addressing the escalating demands of DASCs research. The development of cutting-edge characterization methodologies is therefore not only a pivotal prerequisite for overcoming the bottlenecks in fundamental DASCs research, but also a critical safeguard for facilitating their translation from laboratory-scale fundamental studies to practical applications, such as fuel cells and ZABs. For the atomic-level structural elucidation, electronic structure characterization, and catalytic mechanism verification of DASCs, the advanced characterization techniques reported to date can be categorized into three major classes (Fig. 11). (1) Atomic-level microstructural characterization techniques for confirming the existence and coordination environments of dual-atom sites [21], including aberration-corrected high-angle annular dark-field scanning transmission electron microscopy (AC-HAADF-STEM), X-ray absorption fine structure (XAFS) spectroscopy, and scanning tunneling microscopy (STM); (2) Electronic and chemical environment characterization techniques for unraveling the synergistic interaction mechanisms of dual-atom sites [3], encompassing X-ray photoelectron spectroscopy (XPS), electron paramagnetic resonance (EPR) spectroscopy, and Mössbauer spectroscopy; (3) In situ/operando characterization techniques for dynamically probing the catalytic processes and site stability [20], such as in situ Raman spectroscopy, in situ Fourier transform infrared (FTIR) spectroscopy, and operando electrochemical mass spectrometry (EC-MS).

### Aberration-Corrected High-Angle Annular Dark-Field Scanning Transmission Electron Microscopy

HAADF-STEM represents a core imaging mode of STEM, which enables selective imaging of heavy atoms in materials by collecting high-energy electrons scattered from the sample via a high-angle annular detector. Conventional HAADF-STEM, however, is inherently limited by the spherical aberration of electron lenses, which restricts its spatial resolution to above 1 Å and thus renders it inadequate for the atomic-level characterization of single-atom and dual-atom sites. To surmount this critical limitation, the integration of a multipole electromagnetic corrector into the STEM system allows for real-time compensation and correction of spherical aberration in electron lenses. This breakthrough advances the spatial resolution of HAADF-STEM to below 0.5 Å [147], achieving atomic-level resolution and establishing it as the standard technique for characterizing single-atom sites, dual-atom sites, and atomic-scale microstructures in contemporary materials science and catalytic chemistry research. The imaging principle of AC-HAADF-STEM is based on Rutherford elastic scattering and the *Z*-contrast effect, with its core imaging rule being that the intensity of high-angle scattered electrons is proportional to the square of the atomic number (*Z*^2^). Specifically, in AC-HAADF-STEM images, heavy atoms (e.g., metal atoms such as Fe, Mn, and Cu) appear as bright, discrete dots, whereas light atoms (e.g., C, N, and O) produce negligible signals, forming a dark background. This unique characteristic empowers AC-HAADF-STEM to directly and clearly visualize the bright dots corresponding to dual-atom sites against the dark background of carbon supports, enabling the precise atomic-level localization and distribution analysis of such sites.

**Fig. 12:**
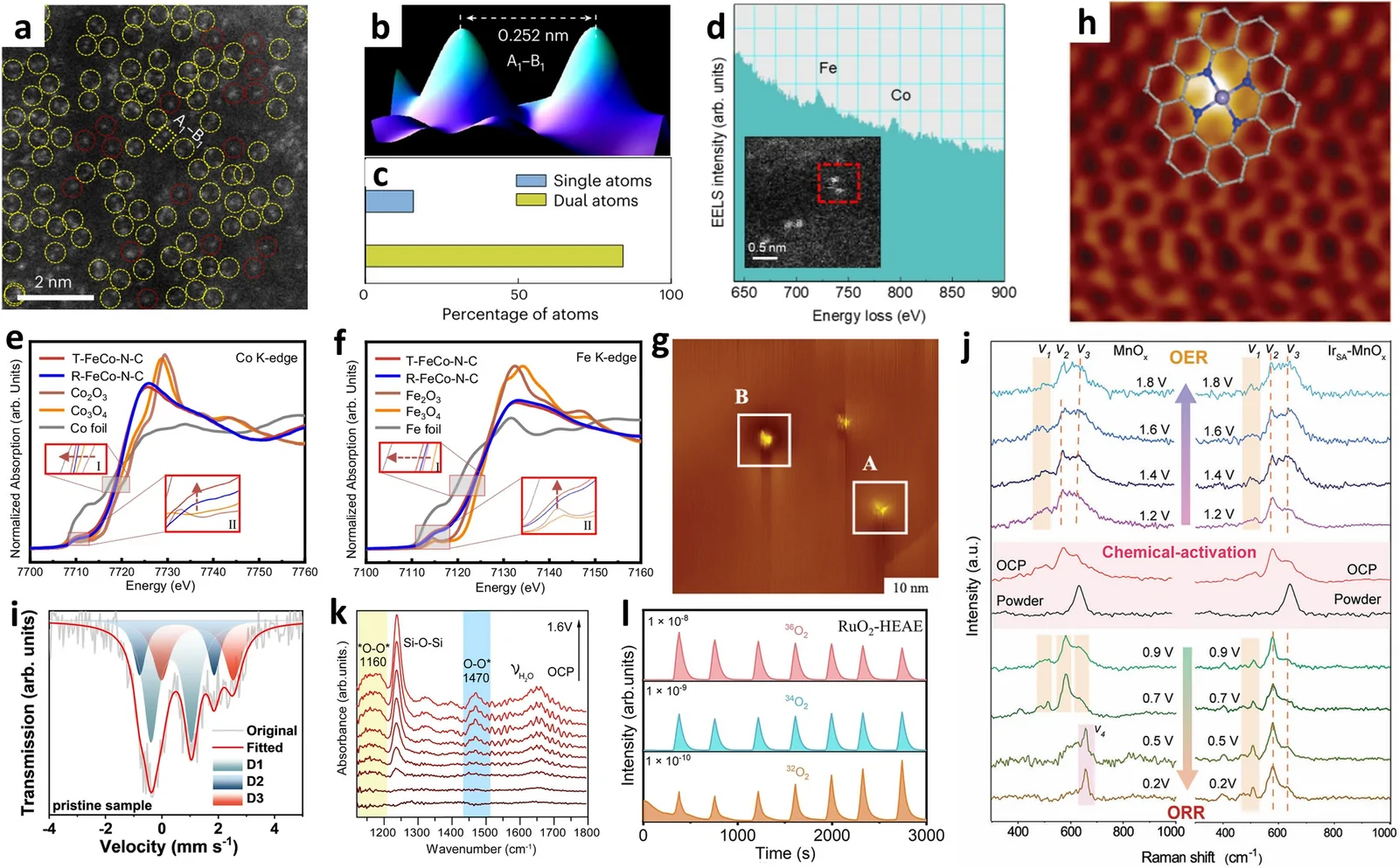
Characterization techniques. **a–c** AC-HAADF-STEM images of FeCo-N_3_O_3_@C. **d** EELS of FeCo-N_3_O_3_@C. Reproduced with permission [56]. Copyright 2024, Springer Nature. **e** Co K-edge XANES. **f** Fe K-edge XANES Reproduced with permission [58]. Copyright 2025, Springer Nature. **g** STM topographic image Reproduced with permission [155]. Copyright 2012, American Physical Society. **h** A low-temperature STM image. Reproduced with permission [156]. Copyright 2015, American Association for the Advancement of Science. **i** ^57^Fe Mössbauer spectrum. Reproduced with permission [158]. Copyright 2025, Springer Nature. **j** In situ Raman spectra. Reproduced with permission [162]. Copyright 2024, Wiley-VCH. **k** In situ FTIRs spectra. Reproduced with permission [164]. Copyright 2024, Published by PNAS. **l** DEMS signals of ^32^O_2_, ^34^O_2_ and ^36^O_2_ from the gaseous products. Reproduced with permission [165]. Copyright 2025, Springer Nature

In summary, compared with conventional characterization techniques (e.g., conventional TEM) and other STEM imaging modalities, AC-HAADF-STEM exhibits three distinct core advantages as follows [148]. (1) Atomic-scale spatial resolution: The incorporation of spherical aberration correction technology enables a spatial resolution of below 0.5 Å, allowing for the direct visualization of the adjacency of two metal atoms in dual-atom sites (e.g., the interatomic distance of the dual-atom pair); (2) Atomic number-dependent selective contrast: Leveraging its intrinsic imaging principle, AC-HAADF-STEM enables the precise discrimination of metal atoms with different atomic numbers; (3) Nondestructive imaging: Since AC-HAADF-STEM relies on elastically scattered electrons for imaging, it induces minimal damage to the sample. This allows for repeated scanning and high-resolution imaging of the same region, thereby ensuring the reliability and reproducibility of the characterization results. For instance, Wang et al. synthesized a Janus-type DASC, FeCo-N_3_O_3_@C [56], in which the bimetallic centers are bridged by N and O atoms. The authors employed AC-HAADF-STEM imaging to confirm the coexistence of a small fraction of single atoms and a high abundance of dimers in this catalyst (Fig. 12a–c). Statistical analysis revealed that dual-atomic pairs account for 85% of all identifiable bright spots. Further statistical characterization of 12 dual-atomic pairs demonstrated that the interatomic distance between adjacent metal centers is approximately 2.5 Å, which is comparable to the typical bond length of metal–metal bonds in metallic phases (~ 2.5 Å), indicative of the formation of direct metal–metal bonds between the paired atoms. In addition, electron energy loss spectroscopy (EELS) measurements performed on the highlighted regions featuring paired bright spots in the inset of the AC-HAADF-STEM image of FeCo-N_3_O_3_@C (Fig. 12d) provided unambiguous evidence for the formation of Fe-Co dimers. Chen et al. constructed an Fe-Nb ADASC (FeNb/C-SNC) by incorporating Nb and S atoms into a concave cubic carbon support derived from MOFs [64]. AC-HAADF-STEM analysis revealed that the Fe and Nb species are atomically dispersed on the carbon support. Statistical analysis of the interatomic distance distribution confirmed that the distance between most paired sites is approximately 2.6 Å. Collectively, the characterization results obtained via AC-HAADF-STEM demonstrated that the authors have successfully synthesized the Fe-Nb dual-atomic-pair catalyst.

Despite its exceptional capability for precisely characterizing the distribution of heavy atoms, AC-HAADF-STEM fails to resolve the coordination environments of light atoms (e.g., C and N) [149]. This intrinsic limitation precludes the comprehensive elucidation of the full coordination configurations of dual-atom sites. To bridge this critical knowledge gap, integrated differential phase contrast STEM (iDPC-STEM) has emerged as a complementary imaging technique. Wei et al. employed integrated differential phase contrast scanning transmission electron microscopy (iDPC-STEM) to directly visualize the adsorption and desorption behaviors of pyridine and thiophene molecules within ZSM-5 zeolites under in situ atmospheric conditions [150]. This advanced characterization technique enabled atomic-resolution imaging of individual pyridine and thiophene molecules confined within the channels of ZSM-5 zeolites, thereby achieving the real-space visualization of the strong interactions between these probe molecules and the acidic active sites. When employed in correlative analysis with AC-HAADF-STEM, it enables the simultaneous visualization of heavy atom distributions and light atom coordination environments. This synergistic characterization strategy thus facilitates the comprehensive decoding of the atomic configurations of dual-atom sites and their local coordination environments in dual-atom catalysts, thereby furnishing the most direct and comprehensive experimental evidence for the existence of dual-atom sites. For instance, Gan et al. employed AC-HAADF-STEM in conjunction with state-of-the-art iDPC-STEM to directly visualize interstitial hydrogen atoms within the 1–3 subsurface atomic layers of Pt-based nanoparticles [151]. This pivotal observation revealed that in situ formed platinum surface hydrides optimize the adsorption characteristics of hydrogen intermediates, which in turn significantly enhances catalytic activity and enables the achievement of a self-regulated hydrogen electrocatalytic process.

### X-Ray Absorption Fine Structure Spectroscopy

XAFS spectroscopy is an element-selective, atomic-scale structural characterization technique based on synchrotron radiation sources, which enables the comprehensive analysis of the oxidation state, local coordination environment, bond length, coordination number, and interatomic interactions of specific elements within materials [152]. A defining hallmark of XAFS lies in its exceptional element specificity: By tuning the excitation energy to the characteristic absorption edge of the element of interest, interference from other elements can be effectively mitigated. This unique attribute renders XAFS particularly robust for the precise characterization of metal active sites in complex catalytic systems. Depending on the energy range relative to the absorption edge, XAFS can be further divided into two complementary sub-techniques: X-ray absorption near-edge structure (XANES) and extended X-ray absorption fine structure (EXAFS) spectroscopy.

The oscillatory signals of XANES originate from the multiple scattering of photoelectrons and the resonant absorption of the electronic DOS, with its core information encompassing two aspects. (1) The position of the absorption edge: The energy shift of the absorption edge is directly correlated with the oxidation state of the target element. A higher oxidation state leads to a stronger binding energy between inner-shell electrons and the atomic nucleus, requiring a higher excitation energy and thus causing the absorption edge to shift toward higher energies; conversely, a lower oxidation state results in a shift of the absorption edge toward lower energies. For instance, Yuan et al. reported a Fe-Co DACs featuring a Janus effect [58]. XANES measurements revealed that the absorption edges of Co and Fe in T-FeCo-N-C are shifted toward lower energy relative to those in R-FeCo-N-C, which are close to the characteristic edges of the + 2 oxidation state (Fig. 12e, f). Furthermore, owing to the subtle difference in electronegativity between Co (1.88) and Fe (1.83), considerable charge transfer occurs at both Fe and Co atomic sites in T-FeCo-N-C. This observation not only attests to the strong intrinsic electronic interactions between Fe and Co atoms but also provides direct experimental evidence for the electronic synergy of dual-atomic sites. (2) The shape and oscillatory features of the absorption edge: These reflect the coordination symmetry and electronic structure of the target element. Furthermore, the charge transfer between metal atoms in dual-atomic sites can also induce variations in the intensity of characteristic peaks in the XANES spectra. For instance, Zeng et al. constructed an FeCo-N/P–C ADASC capable of addressing the critical issue of the ^*^OH/^*^OOH scaling relationship in the ORR [153]. XANES characterization at the Fe K-edge and Co K-edge revealed a significant enhancement in the intensity of the pre-edge peak, which was attributed to the 1*s* → 3*d* electronic transition induced by P doping. This observation indicates that the structural symmetry around the Fe and Co atoms in FeCo-N/P–C is substantially disrupted, thus confirming the asymmetric architecture of the catalyst.

The oscillatory signals of extended X-ray absorption fine structure (EXAFS) arise from the single and multiple scattering processes of photoelectrons, whose fundamental principle can be elucidated by the free electron model and backscattering theory. When the photon energy exceeds that of the absorption edge, the excited photoelectrons propagate outward as spherical waves. Upon encountering adjacent surrounding atoms (e.g., ligand atoms), these photoelectrons undergo backscattering, generating backscattered waves. The backscattered waves then interfere with the incident spherical waves at the atomic nucleus: When the wavelength of the photoelectrons satisfies specific conditions, constructive interference occurs, leading to an increase in the absorption coefficient; in contrast, destructive interference results in a decrease in the absorption coefficient. This interference process exhibits periodic oscillations as a function of photon energy, which constitutes the EXAFS signal. By performing Fourier transformation on the EXAFS signal, a radial distribution function (RDF) can be obtained, where the abscissa represents the interatomic distance (Å) and the ordinate denotes the intensity of the backscattering signal. Fitting and analysis of the characteristic peaks in the radial distribution function enable the extraction of critical structural parameters, including the coordination number, bond length, and structural disorder. For instance, in their investigation of the coordination environment of as-synthesized Fe-Sn dual-atomic-pair sites, Zhang et al. employed EXAFS spectroscopy to resolve the characteristic signals of Fe–N, Fe–Sn, and Sn–N bonds in FeSnNC [55], thereby confirming the atomic dispersion of Fe and Sn species. Quantitative least squares EXAFS fitting further demonstrated that each Fe atom in FeSnNC is coordinated to four N atoms and one Sn atom, whereas each Sn atom is coordinated to four N atoms. Collectively, these results unequivocally validate the successful formation of FeN_4_-SnN_4_ dual-atomic-pair sites within the FeSnNC catalyst.

However, XAFS yields only the average structural information of the target element, and cannot provide spatial distribution images of individual atoms, as is achievable by AC-HAADF-STEM. For this reason, a combination of XAFS and AC-HAADF-STEM is typically required to realize the complementarity between average structural information and atomic-scale spatial distribution imaging. Furthermore, XAFS can be coupled with equipment such as electrochemical workstations and reaction cells to enable in situ/operando characterization, which allows for the real-time monitoring of the structural evolution of metal active sites under realistic reaction conditions. This thereby provides dynamic data crucial for elucidating catalytic mechanisms and deactivation pathways.

### Scanning Tunneling Microscopy

Scanning tunneling microscopy (STM) represents one of the core techniques within the scanning probe microscopy (SPM) family. Its defining characteristic lies in the raster scanning of a sample surface on a point-by-point basis using an ultra-sharp probe tip, enabling atomic- or nanoscale characterization of material surfaces [154]. Operating on the principle of the quantum tunneling effect, STM is restricted to the characterization of electrically conductive samples, yet it can provide atomic-resolution information regarding surface topography and local electronic structure. In the context of atomically dispersed catalytic materials research, although STM cannot directly characterize bulk-phase dual-atomic sites in the same manner as AC-HAADF-STEM or XAFS spectroscopy, it offers unique capabilities in model catalyst systems (e.g., atomically dispersed sites supported on single-crystal surfaces). Specifically, STM enables atomic-level localization of surface atomic sites, coupled with the characterization of their electronic structures and analysis of surface interactions, thereby furnishing fundamental experimental evidence for deciphering the synergistic catalytic mechanisms of dual-atomic sites. For instance, Nakamura et al. employed STM to investigate the electronic interactions between N dopants and the surrounding C atoms in N-doped graphene, as illustrated in Fig. 12g [155]. Additionally, Bao et al. utilized low-temperature STM to probe the electronic structure of individual FeN_4_ moieties supported on a graphene matrix, as depicted in Fig. 12h [156]. In this characterization, the Fe centers appear as distinct bright spots, while the adjacent atoms exhibit a lower apparent contrast compared to other carbon atoms in the graphene matrix. Furthermore, STM simulations were performed to reconstruct the electronic structure of the FeN_4_ centers, which exhibited excellent agreement with the experimentally obtained STM images.

In practical research, a single characterization technique fails to comprehensively characterize DASCs. For real catalyst systems, a combined technical strategy integrating STM/AFM, AC-HAADF-STEM, and XAFS is typically adopted. This integrated approach enables complementary investigations of model and real catalyst systems, thereby furnishing comprehensive and robust experimental evidence for the synergistic catalytic mechanisms, catalytic performance, and stability of dual-atomic-site catalysts.

### Electron Paramagnetic Resonance and Mössbauer Spectroscopy

EPR and Mössbauer spectroscopy are both selective characterization techniques tailored for probing specific microstructures and electronic states, and they exhibit high complementarity in the study of catalytic materials [157]. Also known as electron spin resonance (ESR) spectroscopy, EPR is based on the spin resonance phenomenon of unpaired electrons. This technique enables qualitative and quantitative analysis of paramagnetic species in materials (e.g., transition metal ions, free radicals, and defect sites), while providing critical insights into their electronic spin states, coordination environments, and intermolecular/interatomic interactions. In contrast, Mössbauer spectroscopy relies on the recoil-free resonant absorption of γ-rays. To date, this technique has been applicable to nearly 100 isotopes of over 40 elements, among which ^57^Fe represents the most widely used Mössbauer-active nuclide. Consequently, Mössbauer spectroscopy has emerged as a technique uniquely suited for the characterization of Fe-based catalytic materials, allowing for precise determination of the oxidation state, spin state, coordination environment, chemical bonding geometry, and interatomic interactions of Fe atoms.

Consequently, in the investigation of Fe-M DASCs, the synergistic integration of EPR and Mössbauer spectroscopy enables precise characterization spanning from electronic spin states to Fe-specific local coordination environments. Specifically, EPR spectroscopy facilitates comprehensive profiling of paramagnetic species within dual-atomic sites (e.g., Fe ions, Mn/Cu ions, and free radicals), thereby yielding holistic insights into the overall electronic spin configuration of the catalytic system. Mössbauer spectroscopy, on the other hand, can perform atomically resolved, Fe-exclusive fine structural analysis, directly distinguishing the differences between dual-atomic sites, single-atomic sites, and clusters, and revealing the coordination environments of Fe atoms. For instance, Xing et al., in their structural investigation of Fe–Mn bimetallic single-atom catalysts [158], employed ^57^Fe Mössbauer spectroscopy to confirm that iron species predominantly exist as single-atom Fe(II) and Fe(III) species (Fig. 12i), thus ruling out the formation of metallic clusters. Separately, Xue et al. integrated EPR with ^57^Fe Mössbauer spectroscopy to elucidate that the iron species in the as-synthesized PtFe@Fe_SAs_-N–C catalyst exist in both single-atom and alloy forms [159], with the single-atom component being dominated by high-spin Fe^3+^ species. The integration of EPR and Mössbauer spectroscopy enables precise characterization, ranging from a holistic to a local perspective and from a universal to a specific level, thereby providing robust experimental evidence for the existence of dual-atomic sites.

### In Situ/Operando Characterization Techniques

In the study of DASCs, conventional ex situ characterization techniques only provide static structural information before and after the reaction, failing to elucidate the dynamic processes catalytic reactions and their deactivation mechanisms. By contrast, the synergistic combination of in situ Raman spectroscopy, in situ FTIR spectroscopy, and operando EC-MS enables full-process dynamic characterization, ranging from catalyst structural evolution and intermediate adsorption/transformation to product formation [160]. This integrated analytical strategy not only delivers direct and robust experimental evidence for the synergistic catalytic mechanisms, anti-demetallation stability, and deactivation mechanisms of dual-atomic sites but also serves as an indispensable suite of dynamic characterization techniques in current investigations.

In situ Raman spectroscopy operates on the principle of the Raman scattering effect of molecular vibrations. It is primarily utilized to characterize the carbon support structure of catalysts (evidenced by the variations in the D, G, and 2D bands) as well as the reaction intermediates during catalysis (e.g., the vibrational band of the O–O bond) [161]. Furthermore, this technique can be employed to monitor the structural stability of dual-atomic sites during cyclic operations and to elucidate the reaction pathways of oxygen electrocatalytic reactions. For instance, Peng et al. employed in situ Raman spectroscopy to investigate the ORR and OER processes of Ir_SA_-MnO_x_ [162], as well as their distinct reaction mechanisms, thereby unraveling the origin of its high catalytic activity. As illustrated in Fig. 12j, during the ORR process, the structure of the MnO_6_ units in Ir_SA_-MnO_x_ remained nearly unchanged, whereas MnO_x_ exhibited significant band shifts. This observation supports the existence of distinct reaction pathways for the catalyst in ORR and OER. Combined with in situ XAFS characterization, further studies confirmed that the high-valence Ir sites stabilize the Mn^3+^/Mn^4+^ active sites, enabling them to maintain structural stability under high-potential conditions and thus enhancing the overall catalytic activity and stability. In situ FTIR spectroscopy operates based on the infrared absorption effect of molecular vibrations. Endowed with ultra-high sensitivity and selectivity toward adsorbed intermediates, this technique enables real-time detection of species adsorbed on active sites during catalytic reactions (e.g., ^*^OOH, ^*^O, and ^*^OH adspecies) [163]. By analyzing the shifts in peak positions and variations in peak intensities of these intermediates, it can elucidate the catalytic mechanisms of dual-atomic sites—for example, verifying whether the 4e^−^ pathway dominates the ORR. Among its various modalities, attenuated total reflection FTIR (ATR-FTIR) spectroscopy is particularly suitable for in situ characterization in electrolyte environments, thus emerging as a widely utilized technique in electrochemical catalysis research. For instance, Tian et al. employed ATR-FTIR spectroscopy (Fig. 12k) to demonstrate that the catalytic mechanism of the as-synthesized stereo-Fe–Co DSC and planar-Fe–Co DSC does not follow the conventional adsorption evolution mechanism (AEM) or lattice oxygen mechanism (LOM) [164]. Instead, it proceeds via a dual-site mechanism, also referred to as the oxide pathway mechanism (OPM). Separately, Peng et al. utilized in situ attenuated total reflection surface-enhanced infrared absorption spectroscopy (ATR-SEIRAS) to reveal that Ir_SA_-MnO_x_ forms Mn=O species and ^*^–O–O–^*^ bridged oxygen species at a potential of 1.4 V [162]. This observation confirms that the OER pathway is dominated by direct O–O coupling, rather than the traditional AEM mechanism. Operando EC-MS relies on the mass-to-charge ratio analysis of catalysts via mass spectrometry. This technique enables the online, real-time detection of gaseous or liquid products generated during electrocatalytic reactions, while allowing for the accurate quantification of product selectivity, formation rate, and electron transfer number. As such, it has established itself as a standard technique for validating electrocatalytic reaction pathways and catalyst stability. For instance, Song et al. employed in situ differential electrochemical mass spectrometry (DEMS) (Fig. 12l) combined with ATR-SEIRAS to demonstrate that RuO_2_-HEAE follows the OPM rather than the conventional AEM [165]. This enables direct O–O coupling and avoids the formation of the ^*^OOH intermediate.

Overall, in situ Raman spectroscopy, in situ FTIR spectroscopy, and operando EC-MS exhibit high complementarity in characterization, collectively forming a technical closed loop for dynamic analysis. This integrated framework enables comprehensive and correlated investigations through the following synergistic roles. (1) In situ Raman spectroscopy: Provides dynamic information on the bulk structure of catalysts, reflecting their structural stability and reconstruction behavior during reactions. (2) In situ FTIR spectroscopy: Offers insights into the adsorption and transformation pathways of adsorbed intermediates, unraveling the microcosmic mechanisms underlying catalytic reactions. (3) Operando EC-MS: Delivers quantitative data on the formation rate and selectivity of reaction products, validating the macroscopic reaction pathways and the overall catalytic performance of catalysts.

Advanced characterization techniques are pivotal for advancing DASCs from empirical synthesis toward precise design. Their continuous development and advancement enable the overcoming of atomic-level characterization bottlenecks, establishing structure–activity relationships, unraveling dynamic mechanisms, and addressing both fundamental research needs and application-driven requirements. The synergy between static and dynamic characterization methodologies facilitates comprehensive, multi-dimensional elucidation of catalysts, thereby fostering mechanistic innovation and translational research. Nevertheless, inherent challenges persist, including incomplete information derived from individual techniques, heavy reliance on sophisticated instrumentation, intricate data interpretation, and intrinsic limitations specific to each characterization approach. To surmount these existing bottlenecks, it is imperative to further strengthen technical synergy and data fusion, promote equipment upgrading and innovation, and optimize sample preparation and characterization protocols.

## ADASCs for Efficient Oxygen Electrocatalysis

Electrochemical catalysis offers a promising strategy for sustainable energy conversion due to its high selectivity and mild operating conditions. Among electrochemical reactions, oxygen electrocatalytic reactions (ORR/OER) are core half-reactions in advanced energy devices such as fuel cells and rechargeable zinc–air batteries [166–168]. These processes involve not only complex mechanistic pathways but also multiple intermediates and other factors, including proton-coupled electron transfer processes and potential-dependent electrode structure changes. The sluggish kinetics of oxygen electrocatalytic reactions constitutes a critical bottleneck for the practical application of related energy devices [169–171]. Conventional SASCs possess uniform active sites, which renders it challenging to simultaneously optimize the adsorption/desorption behaviors of intermediates in ORR and OER and break the inherent LSR. This limitation not only hinders the improvement of catalytic performance but also impedes the widespread commercial application of SASCs and related electrochemical technologies. As an extension of SASCs, DASCs may provide new opportunities for modifying the structure of metal centers and optimizing the adsorption configuration of reactants. Unlike SDASCs—which feature homonuclear metal pairs and symmetric coordination environments—ADASCs stand out due to their unique structural advantages, including asymmetric coordination environments and electronic coupling between heteronuclear metals. These advantages enable ADASCs to break the inherent LSR in ORR/OER, thereby achieving a significant improvement in catalytic performance. Recent studies have shown that the unique configuration of ADASCs is highly effective in regulating electrochemical catalytic activity and stability (Table 1). In the following section, we will elaborate on the application of these unique structural advantages of ADASCs in oxygen electrocatalysis.

**Table 1:** Summary of asymmetric dual-site engineering for enhanced oxygen electrocatalytic performance

Sample	Catalytic reaction	Coordination structure	Electrolyte	Activity	Stability	Refs
Fe-Nb/c-SNC	ORR	Fe–S_1_N_3_/Nb–N_4_	0.1 M KOH	*E*_1/2_, 0.922 V	Stable cycle 5000 cycles	[64]
CoMn-NSC	ORR	CoN_2_S–MnN_3_	0.1 M KOH	*E*_1/2_, 0.901 V	Stable cycle 5000 cycles	[47]
Pt_1_Co_1_/NC-Cl	ORR	Pt(Cl)–Co–N_6_	0.1 M HClO_4_	*E*_1/2_, 0.841 V	Stable cycle 5000 cycles	[91]
Fe, Mn/N–C	ORR	Fe, Mn/N_6_	0.1 M KOH	*E*_1/2_, 0.928 V	Stable cycle 40,000 cycles	[48]
FeSn-C_2_N	ORR	FeN_2_–SnN_2_	0.1 M KOH	*E*_1/2_, 0.914 V	Stable cycle 10,000 cycles	[114]
FeCu-NC	ORR	FeN_4_–CuN_4_	0.1 M KOH	*E*_1/2_, 0.918 V	Stable cycle 5000 cycles	[78]
Fe,W–N-C	ORR	Fe–N_4_/W–N_4_	0.1 M KOH	*E*_1/2_, 0.900 V	Stable operation for 10,000 h	[116]
A-Fe_2_S_1_N_5_/SNC	OER	FeS_1_N_2_–FeN_3_	1.0 M KOH	*η*_10_, 193 mV	Stable operation for 2,000 h	[46]
In-RuO_2_/G	OER	Ru–O-In	1.0 M KOH	*η*_10_, 187 mV	Stable operation for 350 h	[187]
DA-FC-NG	OER	FeN_3_-CoN_3_	1.0 M KOH	*η*_10_, 268 mV	Stable operation for 30 h	[188]
plane-Fe-Co DSC	OER	Fe–N_4_/Co–N_4_	1.0 M KOH	*η*_10_, 190 mV	Stable operation for 160 h	[164]
FeCo-N_3_O_3_@C	ORR and OER	Fe–N_3_/Co–O_3_	0.1 M KOH	ORR: *E*_1/2_, 0.938 V OER: *η*_10_, 298 mV	Stable cycle 10,000 cycles	[56]
Fe/Ni-NC@PDA	ORR and OER	FeN_4_–NiN_3_O	0.1 M KOH	ORR: *E*_1/2_, 0.890 V OER: *η*_10_, 330 mV	Stable operation for 156 h	[178]
FeNi–N–C	ORR and OER	Fe–N_4_/Ni–N_4_	0.1 M KOH	ORR: *E*_1/2_, 0.850 V OER: *η*_10_, 322 mV	–	[104]

### Electrocatalytic Oxygen Reduction Reaction

The ORR has attracted extensive research attention due to its crucial role in electrochemical sustainable energy conversion technologies. According to the research by Wroblowa et al., the ORR process can follow two reaction pathways depending on the number of transferred electrons: either the 2e^−^ pathway (via H_2_O_2_/HO_2_^−^) or the 4e^−^ pathway (via H_2_O/OH^−^). However, the application of energy conversion devices such as fuel cells and metal–air batteries typically relies on the 4e^−^ process, which inherently involves a higher reaction energy barrier for O_2_ activation [172]. Notably, ^*^O_2_ and ^*^OOH intermediates can strengthen their adsorption on bimetallic sites through a bridging mode; this adsorption behavior makes it more likely to achieve direct 4e^−^ transfer in one step via a dissociative pathway (with direct O–O cleavage), thereby bypassing the sluggish kinetics of the ^*^O_2_ → ^*^OOH conversion step [173–175]. Moreover, the cleavage of the O–O bridge bond can further accelerate the ORR rate by facilitating rapid proton-coupled electron transfer to another ^*^O intermediate, which reduces the energy barrier for subsequent reaction steps [39]. Therefore, DASCs exhibit greater potential for realizing the 4e^−^ ORR pathway compared to SASCs. Within the DASCs system, the asymmetric configuration of ADASCs can further amplify the advantages of bridge adsorption. Specifically, the asymmetry, originating from the differentiated electronic state distribution of heterometallic centers or the varying electronegativity of coordinating atoms, enables precise regulation of charge transfer efficiency during bridging adsorption. This regulation not only optimizes the adsorption energies of O_2_ and ^*^OOH but also breaks the LSR that often limits SDASCs. Consequently, ADASCs significantly enhance the 4e^−^ ORR selectivity and kinetic rate [176, 177].

**Fig. 13:**
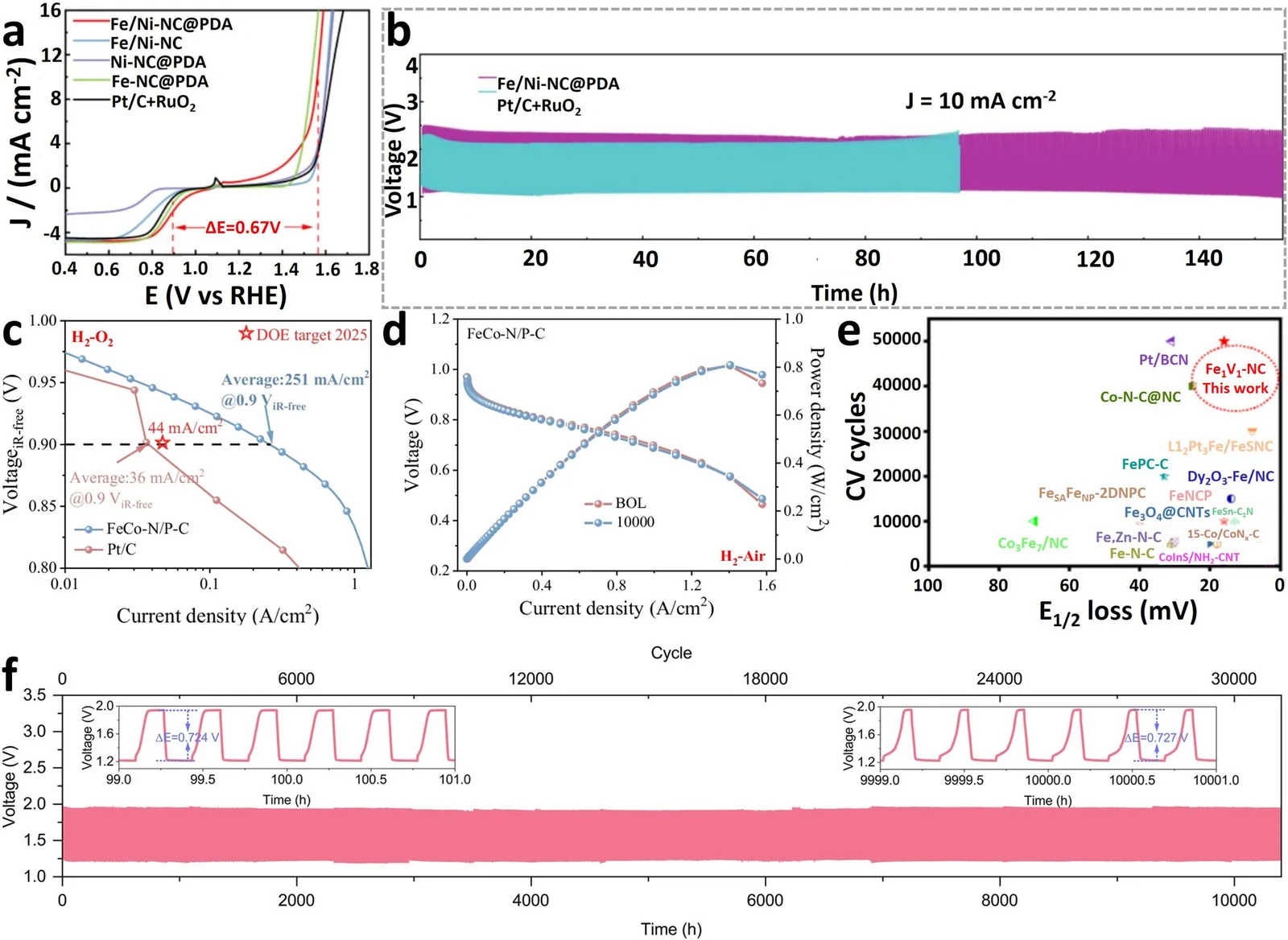
Research on the design and catalytic performance of ADASCs for electrocatalytic ORR. **a** Δ*E* comparison of different catalysts. **b** Charge–discharge cycling curves. Reproduced with permission [178]. Copyright 2025, Elsevier. **c** Tafel plot and **d** H_2_–air fuel cell performance at BOL and EOL of the FeCo-N/P–C. Reproduced with permission [153]. Copyright 2025, Springer Nature. **e** Determination of the catalysts’ activities. Reproduced with permission [179]. Copyright 2025, Wiley-VCH. **f** Charge–discharge cycling curves. Reproduced with permission [116]. Copyright 2025, Springer Nature

Tian et al. prepared a bifunctional oxygen catalyst (Fe/Ni-NC@PDA) with heterogeneous asymmetric bimetallic sites (FeN_4_-NiN_3_O) using NH_2_-MIL-101(Al) as a precursor [178]. Electrochemical tests showed that Fe/Ni-NC@PDA exhibits excellent bifunctional catalytic performance in ORR (∆*E*_1/2_ = 0.89 V) and OER (*η*_j=10_ = 1.56 V), resulting in a potential difference of only 0.67 V. This performance is superior to that of the commercial Pt/C + RuO_2_ catalyst (Fig. 13a). Moreover, in a constant-current charge–discharge cycle test, the catalyst maintained stable cycling for 156 h obvious activity decay, demonstrating its potential for practical device applications (Fig. 13b). DFT calculations revealed that the enhanced ORR/OER performance is attributed to *d–d* orbital coupling between Fe and Ni and their asymmetric electronic structure, synergistically optimizing the adsorption and desorption of the ^*^OOH intermediate. Based on the heterometallic centers in ADASCs, constructing an asymmetric coordination environment via coordinating heteroatoms can synergistically regulate the electronic structure of active sites and the adsorption energy of intermediates, thereby breaking the constraints of the LSR and enhancing catalytic activity and stability. Guided by this concept, Zeng et al. proposed a novel strategy to break the LSR between ^*^OH and ^*^OOH [153]. First, ZIF-8 was prepared as a N-doped carbon precursor, and poly(cyclotriphosphazene-co-p-phenylenediamine) (PZP) was coated on its surface to form a core–shell structure ZIF-8@PZP. Then, Fe-Co-P precursors were encapsulated in ZIF-8@PZP via spatial confinement to obtain FeCo-ZIF-8@PZP, which was subsequently pyrolyzed to synthesize a P-containing Fe-Co dual-atom-site catalyst (FeCo-N/P–C). FeCo-N/P–C achieved a high current density of 251 mA cm^−2^@0.9 V_iR-free_ under 1.5 bar H_2_–O_2_ conditions, far exceeding the U.S. Department of Energy (DOE) 2025 target (44 mA cm^−2^). Under H_2_–air conditions, it delivered a peak power density of 0.805 W cm^−2^ (Fig. 13c). After 10,000 accelerated durability tests, the catalyst showed almost no activity decay, demonstrating excellent structural stability and long-term durability (Fig. 13d). DFT revealed that the introduction of the electron-withdrawing P atoms adjusts the energy levels of heterometallic Fe-Co dual sites, achieving precise matching of the 3*dz*^*2*^ orbital energy levels of Fe and Co. This promotes the ORR along the dissociative pathway, fundamentally avoiding ^*^OOH intermediate formation and bypassing the dual limitations of activity and stability imposed by the traditional LSR. However, due to the similar 3*d* electronic configurations of the second metal (Co) and Fe, the capacity to break the symmetric charge distribution of active sites is constrained. Thus, utilizing heteronuclear metal centers with significant differences in electronic configurations may be more effective in breaking the charge distribution symmetry of diatomic sites composed of a single metal or metals with similar electronic structures. For instance, xiang et al. designed and synthesized an asymmetric Fe-Ru dual-atom catalyst (FeRu-DACs) under the guidance of theoretical calculations [179]. In this catalyst, Ru acts as an electron buffer site: It accepts electrons from Fe and synergistically optimizes the electronic structure of Fe, lowering the *E*_d_ of Fe. FeRu-DACs exhibited excellent ORR performance: Its peak power density reached 1.73 W cm^−2^, an 86% improvement compared to the pristine Fe–N-C catalyst. Moreover, its current density at 0.9 V reached 58 mA cm^−2^, far exceeding the DOE 2025 target (44 mA cm^−2^), realizing a significant enhancement in catalytic activity (Fig. 13e). Li et al. utilized the N_4_ units in phthalocyanine molecules to capture 5*d* transition metal W atoms (scraped off from tungsten carbide grinding balls) [116], thereby constructing a Fe–N_4_/W–N_4_ bimetallic site catalyst (Fe,W–N-C). Leveraging the significant 3*d*–5*d* orbital hybridization between Fe and W, this catalyst effectively optimizes the electronic distribution of Fe–N_4_ sites: The overlapping of Fe 3*d* and W 5*d* orbitals weakens the adsorption strength of ^*^OH on Fe, facilitating O_2_ activation and ^*^OH desorption. Meanwhile, the unsaturated 5*d* orbitals and tunable oxidation states of W atoms form a stable coordination network with adjacent N atoms, suppressing Fe site leaching. Zinc–air batteries assembled with Fe,W–N-C exhibited outstanding performance: They achieved a cycle stability exceeding 10,000 h and an energy density of 953 Wh kg^−1^, far surpassing those of commercial Pt/C + IrO_2_-based batteries (Fig. 13f).

It is worth noting that ADASCs not only exhibit excellent ORR catalytic activity in alkaline electrolytes, even surpassing that of commercial Pt/C catalysts, but also, when used as cathodes in zinc–air batteries, outperform commercial Pt/C and achieve a breakthrough in peak power density. Despite their potential for enhancing electrocatalytic performance, the peak power density of ADASCs often fails to exceed that of commercial Pt/C in more complex acidic PEMFCs. This phenomenon is mainly attributed to two aspects: first, precise control over heteronuclear metal centers and asymmetric coordination environments remains challenging. This lack of control tends to cause uneven distribution and low density of active sites, thereby limiting further improvements in catalytic efficiency. Moreover, existing synthesis processes cannot meet the requirements for precise regulation and preparation, leading to a significantly faster activity decay rate in practical applications than that of an ideal ADASC system. Second, the performance of ADASCs in acidic electrolytes is typically inferior to that in alkaline electrolytes—a prevalent challenge across the entire electrocatalyst system, primarily due to the harsher acidic environment. In summary, the stability of ADASCs in the electrocatalytic ORR and PEMFC applications still requires further improvement, stemming primarily from the following four degradation mechanisms: (1) Dynamic instability mechanism of active sites. Under the PEMFC operating conditions (low pH, high potential, and dynamic load fluctuations), the metal–metal covalent bonds at the heteronuclear metal centers of ADASCs are prone to electrochemical cleavage, triggering migration and agglomeration of metal atoms. Concurrently, the asymmetric coordination environment is susceptible to destruction in the strong oxidative atmosphere, leading to the structural collapse of active sites and leaching of metal atoms, which results in an irreversible decline in the number of active sites. (2) Intermediate adsorption imbalance and active site poisoning mechanism. During the ORR process, the adsorption strength of ADASCs toward key intermediates (e.g., ^*^OOH, ^*^O, ^*^OH) tends to deviate due to insufficient precision in d-band center modulation. Excessively strong adsorption clogs the active sites and induces their structural reconstruction, whereas excessively weak adsorption impairs the activation efficiency of reactants, exacerbating side reactions (e.g., H_2_O_2_ generation) that further erode the active sites. (3) Interface and support degradation mechanism. Insufficient metal–support interactions (MSI) cause interfacial electron imbalance during PEMFC startup–shutdown cycles, which induces oxidative corrosion of supports such as carbon-based materials. Meanwhile, poor water management within the membrane electrode assembly accelerates electrolyte permeation, undermining the interfacial compatibility between ADASCs and the proton exchange membrane, and thus leading to the overall performance degradation of the catalyst. (4) Insufficient operating condition adaptability mechanism. Fluctuations in load and temperature during practical PEMFC operation disrupt the synergistic effect between the spin configuration and energy level matching of ADASCs, reducing the efficiency of directional electron transfer. Additionally, the weak adhesion between the catalyst and electrode slurry causes catalyst detachment under fluid scouring, resulting in the loss of catalytic functionality. To address the aforementioned stability challenges, four targeted strategies can be adopted as follows: (1) Active site structural reinforcement strategy. Construct ligand-bridged heteronuclear metal covalent bond structures, and enhance the structural rigidity of active sites by virtue of electronic coupling effects. Precisely regulate the interatomic spacing of dual-atom sites to synergistically suppress metal migration and agglomeration through steric hindrance and electronic attraction. Introduce doping with metals stable in high oxidation states to improve the corrosion resistance of active sites. (2) Precise electronic configuration modulation strategy. Optimize spin coupling and strain effects to tune the d-band center to the optimal range for ORR, which matches the adsorption–desorption kinetics of reaction intermediates. Strengthen electron delocalization via *p*–*d*/*d*–*d* orbital hybridization to avoid active site poisoning and structural reconstruction. (3) Interface and support modification strategy. Adopt co-modification of carbon supports with multiple heteroatoms or coating with highly stable metal oxides (e.g., TiO_2_) to enhance metal–support interactions. Optimize the membrane electrode preparation process to improve the adhesion between the catalyst and electrode slurry, thereby enhancing water management capability. (4) Operating condition adaptability optimization strategy. Design adaptive electron transport pathways to improve the tolerance of catalysts to load fluctuations and temperature variations. Develop anti-scouring catalyst coating technologies to minimize catalyst detachment under dynamic operating conditions. Therefore, investigating the reaction mechanisms and deactivation principles of ADASCs under practical ORR operating conditions will be a core task for guiding the development of high-performance catalysts.

### Electrocatalytic Oxygen Evolution Reaction

OER is a pivotal half-reaction in energy conversion and storage systems, such as water splitting for hydrogen generation, metal–air batteries, and regenerative fuel cells. Its catalytic performance directly governs the hydrogen production efficiency and cost, as well as the energy density, cycle stability, and advancement in practical applications of these devices [180–182]. Additionally, OER is a sophisticated 4e^−^ reaction encompassing multiple proton-coupled electron transfer steps. It proceeds via the generation and transformation of a series of intermediates (including ^*^OH, ^*^O, and ^*^OOH), features a prolonged reaction route, and requires overcoming energy barriers for each step, thus resulting in sluggish overall reaction kinetics [183, 184]. Therefore, a certain overpotential is usually required to drive water splitting at a desirable rate, which in turn enhances the overall energy utilization efficiency of proton exchange membrane water electrolyzers. Nevertheless, the intrinsic LSR between the OER intermediates (^*^OH, ^*^O, ^*^OOH) restricts the further reduction of this overpotential [185]. Traditional SASCs are limited in their ability to regulate the electronic properties of isolated active sites, making it difficult to simultaneously optimize the adsorption strengths of different oxygen-containing intermediates. SDASCs enhance the synergistic effect between active sites but fail to break the constraints of the LSR, leading to a persistently high energy barrier for the RDS. Therefore, ADASCs—with their asymmetric structure—provide a new approach to overcoming these aforementioned bottlenecks.

**Fig. 14:**
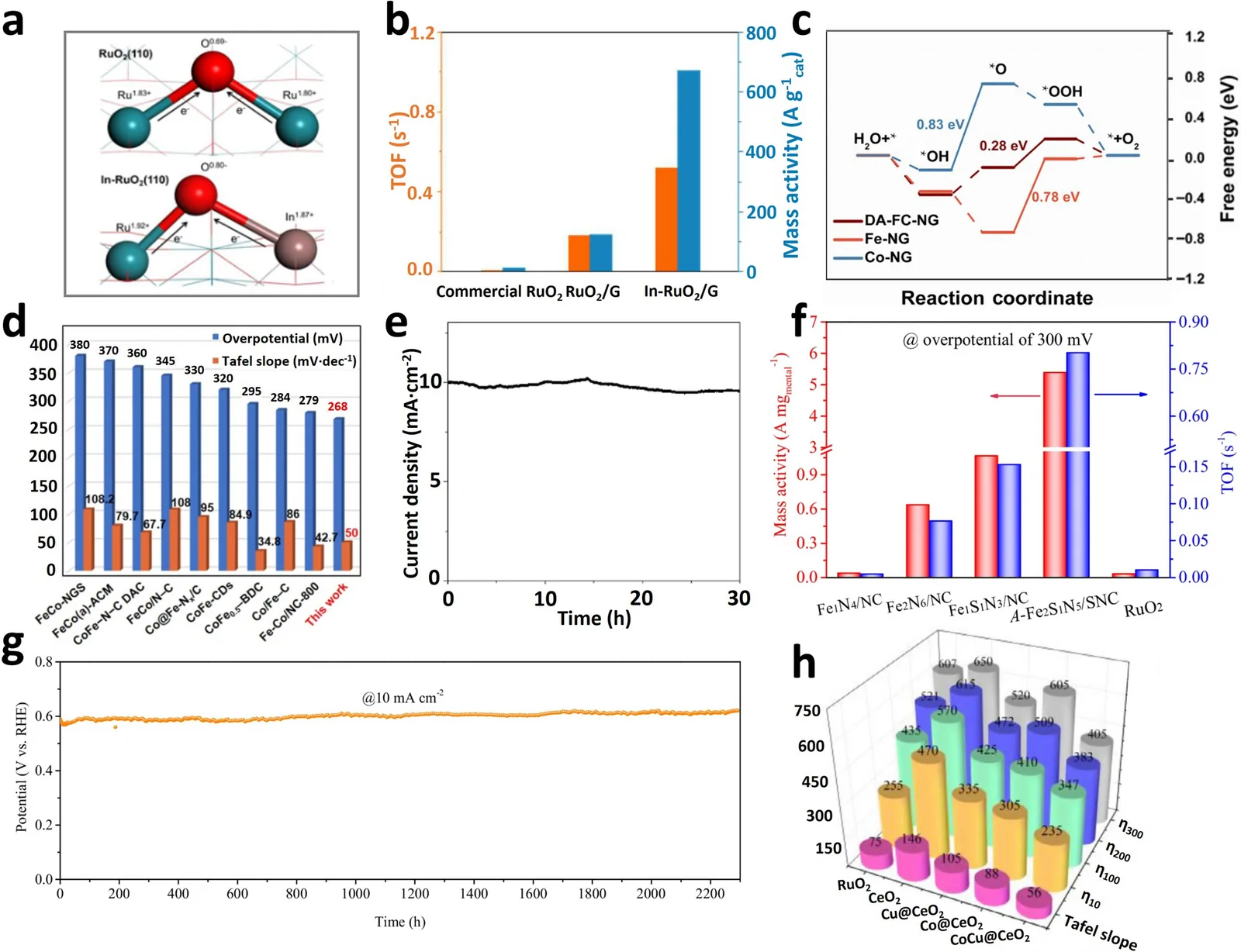
Research on the design and catalytic performance of catalysts for electrocatalytic OER. **a** Comparison of TOF and mass activities. **b** Difference in electron transfer between perfect RuO_2_ and defect In-RuO_2_ described based on Bader charge analysis. Reproduced with permission [187]. Copyright 2023, Wiley-VCH. **c** Δ*G* diagram and **d** comparison of OER activities. **e** Chronoamperometric test of DA-FC-NG. Reproduced with permission [188]. Copyright 2023, Springer Nature. **f** TOF and mass activity and **g** chronoamperometric test of A-Fe_2_S_1_N_5_/SNC. Reproduced with permission [46]. Copyright 2024, Springer Nature. **h** Comparison of overpotential and Tafel slope for different catalysts. Reproduced with permission [163]. Copyright 2025, Wiley-VCH

To minimize the overpotential of the OER, extensive research efforts have been devoted to engineering heterometallic atoms and asymmetric coordination environments to modify ADASCs [186], with the goal of disrupting the symmetric structure, regulating the electronic distribution, and consequently improving catalytic activity and reaction kinetics. Wang et al. employed an ion adsorption strategy, leveraging the confinement of GO to break the traditional Ru–O–Ru symmetry in RuO_2_, and synthesized a catalyst with an asymmetric In-RuO_2_ structure (denoted as In-RuO_2_/G) [187]. Benefiting from the asymmetric Ru–O–In local structural interaction (Fig. 14a), the catalyst exhibits excellent OER performance in acidic media: It delivers a mass activity of 671 A g_cat_^−1^ at 1.5 V, an overpotential of 187 mV at 10 mA cm^−2^, and long-term stability of 350 h at 100 mA cm^−2^ (Fig. 14b). Theoretical calculations revealed that compared with pure RuO_2_, the asymmetric Ru–O–In structure in In-RuO_2_ significantly reduces the formation energy barrier of the ^*^OOH intermediate, thereby inducing a new ^*^OH adsorption RDS. Tang et al. prepared an asymmetric heterometallic atomic catalyst (DA-FC-NG) with an FeN_3_-CoN_3_ configuration via a freeze-drying followed by annealing strategy [188]. DFT calculations indicated that the asymmetric electronic structure interaction between Fe and Co causes charge rearrangement and induces the adsorption of intermediates on the Fe–O–Co bridge structure (Fig. 14c). Endowed with this advantage, the catalyst exhibits excellent OER activity, with a low overpotential (*η*_10_ = 268 mV), outperforming the commercial IrO_2_ catalyst (Fig. 14d, e). Zhang et al. adopted a multilayer stabilization strategy (including defect trapping and coordination riveting) to design a highly defective S, N-co-doped carbon carrier to anchoring Fe–Fe bimetallic sites with an asymmetric coordination environment (A-Fe_2_S_1_N_5_/SNC), with a metal mass loading of 6.72 wt% [46]. Performance evaluation reveals that in alkaline media (1.0 M KOH), A-Fe_2_S_1_N_5_/SNC delivers a current density of 10 mA·cm^−2^ at an OER overpotential of merely 193 mV, outperforming RuO_2_ due to a lower overpotential requirement (Fig. 14f). Moreover, the catalyst can stably electrolyze for over 2000 h at 10 mA cm^−2^, indicating that the durability and catalytic activity of A-Fe_2_S_1_N_5_/SNC can be balanced (Fig. 14g). The enhanced activity of the catalyst is attributed to the synergistic catalytic effect of the high-density active Fe–Fe sites, while its excellent stability stems from the regulation of the metal atom microenvironment by S coordination. The spatial configuration of ADASCs plays a pivotal role in electronic orbital interactions, enabling the optimization of the electronic state of each active site and thus modulating the binding behavior between the sites and catalytic intermediates. Zhang et al. successfully fabricated two asymmetric dual-site catalysts (stereo-Fe-Co DSC and plane-Fe-Co DSC) via distinct electrodeposition approaches [164]. The results indicated that plane-Fe-Co DSC delivers superior OER catalytic performance under alkaline conditions compared to stereo-Fe-Co DSC, with overpotentials of 190 mV (at 10 mA·cm^−2^) and 229 mV (at 100 mA·cm^−2^), respectively. In situ characterizations combined with theoretical calculations revealed that both catalysts follow the oxidation pathway mechanism (OPM). Among them, plane-Fe-Co DSC is more conducive to the dehydrogenation of intermediates in the RDS for O–O bond formation. This work provides a unique perspective on the regulation of ADASCs, and this innovative regulatory strategy is expected to further advance the optimization of dual-atom catalysts, granting asymmetric dual-atom catalysts enhanced performance in future applications. Meanwhile, the rational design of bifunctional electrocatalysts for OER/ORR is crucial for sustainable energy systems. Zhang et al. anchored heterometallic Co–Cu bimetallic sites inside CeO_2_ hollow spheres (denoted as CoCu@CeO_2_) via an effective electrostatic adsorption strategy [163]. The OER mainly occurs at the Co sites, while the Cu sites preferentially promote the ORR. DFT calculations further demonstrated that the long-range synergistic effect, modulated by spatial arrangement and electronic coupling of asymmetric active sites, induces interfacial electronic redistribution and regulates the E_d_, thereby optimizing the binding energies of key intermediates. As a result, the catalyst exhibits a low OER overpotential (235 mV @10 mA cm^−2^) and excellent ORR activity (∆*E*_1/2_ = 0.878 V), surpassing most reported bifunctional OER/ORR catalysts (Fig. 14h).

In summary, ADASCs have made remarkable advances in the electrocatalytic OER. Specifically, synthesized via strategies such as defect trapping and coordination regulation, these catalysts exhibit superior OER performance. This performance advantage stems from heterometallic interactions, which not only optimize the electronic structure of active sites and the adsorption of reaction intermediates but also reduce reaction energy barriers to enhance catalytic activity. Additionally, rational structural design of ADASCs can effectively enhance their stability. Nevertheless, several critical challenges persist regarding the stability of ADASCs in OER and water splitting for hydrogen production applications: (1) Active site structural collapse induced by high-potential strong oxidation. The OER process in water electrolysis for hydrogen production operates at a high potential of 1.5–1.8 V, where the strong oxidative environment readily disrupts the coordination structure of heteronuclear metal centers in ADASCs, leading to the cleavage of metal–ligand bonds. Meanwhile, the excessive elevation of metal oxidation states triggers dissolution and leaching of active atoms, resulting in an irreversible decline in the number of active sites. (2) Intermediate adsorption imbalance and nonselective reconstruction. The adsorption strength of key OER intermediates (e.g., ^*^OH, ^*^O, ^*^OOH) exerts a decisive effect on catalytic performance. Insufficient precision in modulating the *d*-band center and spin configuration of ADASCs tends to cause either overadsorption of oxygen-containing intermediates, which induces nonselective reconstruction of active sites, or under-adsorption, which elevates the reaction energy barrier and exacerbates side reactions (e.g., support oxidation), thereby further eroding the active sites. (3) Metal–support interface degradation and support corrosion. In the case of inadequate MSI, interfacial electron imbalance under high potential accelerates the oxidative corrosion of carbon-based supports (with CO_2_ generation), while oxide supports are prone to phase transition, causing detachment of active sites. Simultaneously, the strongly acidic or alkaline environment of water electrolysis systems intensifies electrolyte permeation across the interface, undermining the integral structure of the catalyst. (4) Performance degradation triggered by dynamic operating conditions and mass transfer limitations. Commercial water electrolyzers operate under dynamic conditions such as current fluctuations and temperature variations, which readily disrupt the synergistic effect between energy level matching and spin coupling in ADASCs, reducing the efficiency of electron transfer. Additionally, the sluggish adsorption and desorption of gas bubbles (O_2_/H_2_) on the catalyst surface block active sites and exacerbate fluid scouring, leading to catalyst exfoliation. To address the aforementioned stability challenges, four targeted approaches can be adopted: (1) Active site structural reinforcement: Construct antioxidative rigid frameworks. (2) Electronic configuration modulation: Match the adsorption kinetics of OER intermediates. (3) Interface and support modification: Enhance corrosion resistance and interfacial compatibility. (4) Operating condition adaptability optimization: Improve dynamic stability and mass transfer efficiency. Meanwhile, future research should also focus on developing facile and scalable synthetic methodologies to simplify the fabrication of ADASCs. Collectively, these endeavors will facilitate the practical application of ADASCs in renewable energy-related energy systems.

## Conclusion and Outlooks

ADASCs are crucial for overcoming the bottlenecks of SASCs (e.g., low active SD and single-dimensional electronic regulation) and SDASCs (e.g., difficulty in adapting to intermediate adsorption in multi-step reactions), significantly enhancing the electrocatalytic performance and stability of energy conversion devices. However, clarifying their complex structure–performance relationships of ADASCs, achieving precise anchoring of heteronuclear metal centers, and realizing controllable construction of asymmetric coordination environments remain critical challenges in current research. This review explores the multi-dimensional performance regulation and key symmetry-breaking strategies of ADASCs. Structurally, heteronuclear metal centers and ligand bridging enhance the anchoring of active sites and inhibit agglomeration; electronically, adjustment of E_d_, spin coupling, and orbital hybridization synergistically optimize active SD and intrinsic activity, providing core support for efficient electrocatalysis. Additionally, the symmetry-breaking strategy focuses on constructing asymmetric structures through heterogenization of metal centers and asymmetric modification of coordination environments. Specifically, heteronuclear metals with significant differences in electronegativity/electronic configuration are introduced, and heteroatom doping or coordination number regulation is used to build asymmetric active sites. These modifications can break the LSR of intermediate adsorption, optimize the adsorption energy of key oxygen electrocatalysis intermediates (^*^O, ^*^OH, ^*^OOH), reconstruct the electronic symmetry of the RDS, and lower the RDS energy barrier. This review systematically analyzes and summarizes the significant value of asymmetric configuration design of ADASCs in oxygen electrocatalysis (ORR/OER), providing insights for the development of high-performance electrocatalysts, while also pointing out the need to deepen research on precise synthesis and dynamic mechanism to promote practical applications.

The synergistic regulation effect of ADASCs not only effectively enhances oxygen electrocatalytic performance but also specifically addresses critical bottlenecks in the field of energy storage batteries. For instance, in lithium–sulfur batteries, this structural advantage enables asymmetric bimetallic sites to simultaneously anchor polysulfides and catalyze their conversion, which efficiently suppresses the dissolution of polysulfides in the electrolyte and the shuttle effect induced by their migration between the cathode and anode, thereby significantly improving battery cycle stability. In sodium-ion batteries, ADASCs similarly leverage this structural superiority to modulate the cathode electronic structure and ion diffusion rates, mitigate volume expansion during charge–discharge cycles, and facilitate their large-scale application as alternatives to lithium-ion batteries. Furthermore, in photocatalysis, this asymmetric dual-site structure promotes the efficient separation of photogenerated charge carriers, inhibits their recombination, and optimizes the adsorption and activation of reaction intermediates, thus substantially enhancing energy conversion efficiency and reaction selectivity in photocatalytic reactions.

**Fig. 15:**
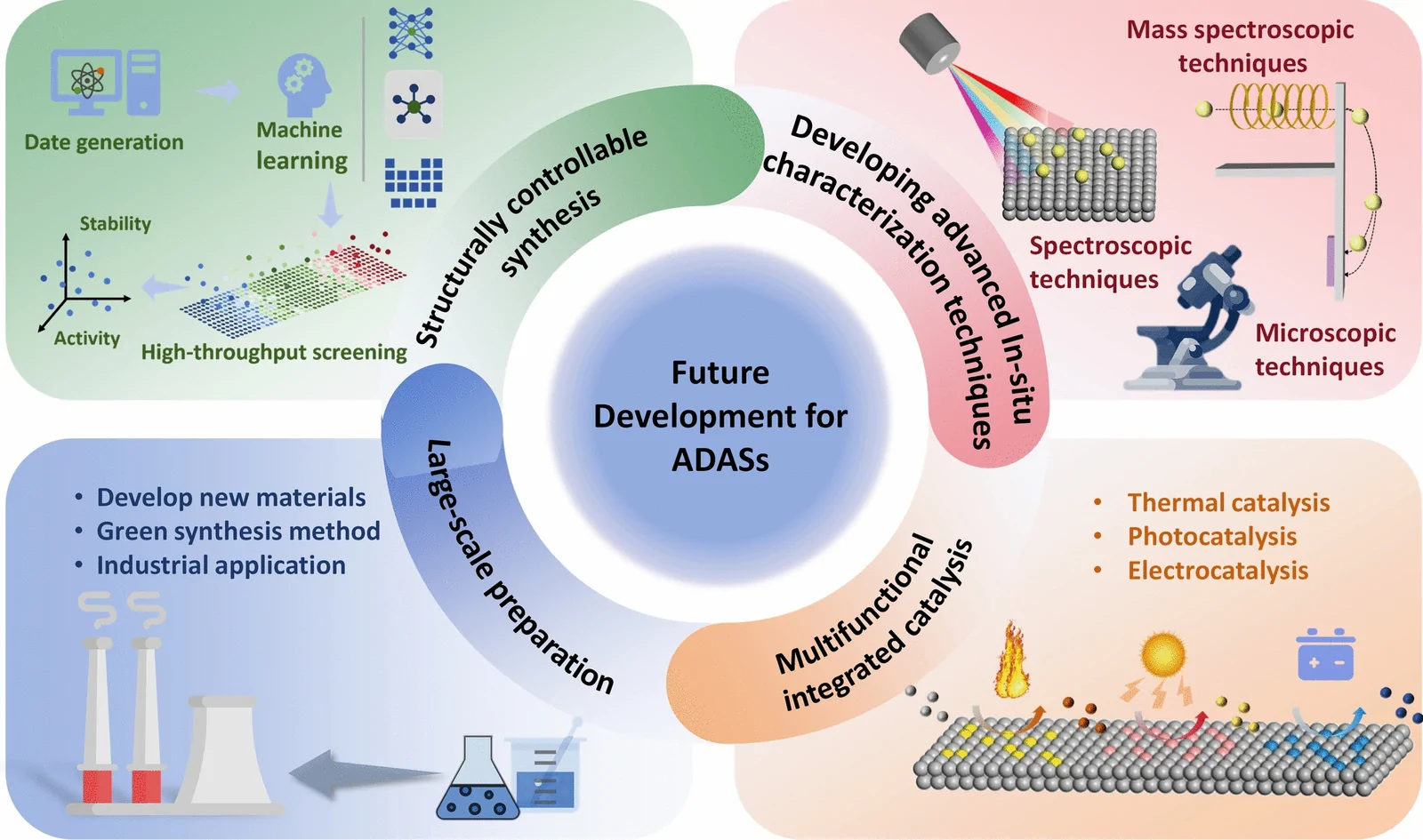
Summary of challenges and opportunities for ADASCs

Although research on ADASCs has achieved some breakthroughs, it is still in its infancy. In the future, the development of ADASCs is likely to focus on key directions such as precise synthesis techniques, multi-functional catalytic integration, and the clarification of dynamic catalytic processes (Fig. 15). Currently, the synthesis of most ADASCs relies on high-temperature pyrolysis steps. Due to the inherent uncertainties of thermodynamic processes during pyrolysis, many unexpected sites are formed, making it challenging to precisely regulate the configuration of active sites. Using cutting-edge synthesis techniques such as atomic-level positioning assembly, high-throughput screening, and dynamic interface regulation, it is expected to achieve precise modulation of asymmetric structures at the sub-nanometer scale. These strategies include regulating the distance between heteronuclear metal atoms, constructing gradient heteroatom coordination, and designing local electron density difference zones in supports, laying a foundation for developing of highly active and stable ADASCs. In addition, green and economical synthesis pathways should be developed to replace traditional processes relying on high-temperature pyrolysis and precious metal precursors. This will reduce preparation costs, mitigate the impact of pyrolysis, and enable the design of continuous production processes to overcome yield limitations of technologies such as ALD—laying the foundation for industrial applications of ADASCs. Meanwhile, multi-functional catalytic integration should be promoted, relying on the advantages of asymmetric structural design to expand its application network and optimize performance. Typically, to address insufficient power density of ADASCs in PEMFCs and poor catalytic activity/durability in acidic systems, it is necessary to deeply explore the corrosion resistance mechanisms of ADASCs in acidic media, develop acid-resistant catalysts, and promote their application in energy conversion devices. Furthermore, to clarify the mechanisms of different ADASCs in various reactions, future research must focus on developing advanced, sensitive, and ultra-high atomic-scale resolution in situ characterization techniques to improve the signal-to-noise ratio and obtain clearer structural and electronic information. Simultaneously, combining theoretical calculations (e.g., high-throughput screening) for guidance can enable more effective structural design and mechanistic studies.

In summary, the asymmetric structural design of ADASCs provides a new paradigm for the development of oxygen electrocatalysts. With breakthroughs in precise synthesis, multi-functional integration, and sustainable preparation technologies, ADASCs are expected to play a transformative role in the field of energy conversion and storage, providing key technological support for solving global energy and environmental issues.
